# Trans-scale hierarchical metasurfaces for multispectral compatible regulation of lasers, infrared light, and microwaves

**DOI:** 10.1515/nanoph-2025-0224

**Published:** 2025-08-01

**Authors:** He Lin, Fuyuan Shen, Zuojun Zhang, Jun Luo, Cheng Huang, Mingbo Pu, Yuetang Wang, Jianping Shi, Xiaoliang Ma, Xiangang Luo

**Affiliations:** National Key Laboratory of Optical Field Manipulation Science and Technology, Institute of Optics and Electronics, Chinese Academy of Sciences, Chengdu 610209, China; School of Automation Engineering, University of Electronic Science and Technology of China, Chengdu 611731, China; College of Materials Sciences and Opto-Electronic Technology, University of Chinese Academy of Sciences, Beijing 100049, China; College of Physics and Electronic Information, Anhui Normal University, Wuhu 241000, China; Research Center on Vector Optical Fields, Institute of Optics and Electronics, Chinese Academy of Sciences, Chengdu 610209, China

**Keywords:** trans-scale hierarchical metasurfaces, RCS reduction, optical transmission enhancement, low-crosstalk imaging, hydrophobic

## Abstract

Electromagnetic scattering control of optical windows has significant challenges in improving optical transmission and compatibility, especially for multispectral and large-angle incidences, due to material and structure mismatches. This paper presents trans-scale hierarchical metasurfaces (THM) to achieve wide-angle optical transmission enhancement and electromagnetic scattering-compatible regulation in dual-band lasers, and infrared and microwave ranges. THM comprises an ultrafine hollow metal array (UHMA) and a transmission-enhanced micro-nanocone array (TMCA). The UHMA regulates microwave radar cross-section (RCS) echo diffuse reflection, while the upper-layer TMCA enables wide-angle optical transmission enhancement. A THM sample of 200 × 200 mm^2^ was fabricated using multistage nanolithography, demonstrating exceptional multifunctional compatibility and optical performance. Results show that the THM sample achieves 10 dB scattering reduction in the 9.5–17.5 GHz microwave band, with average optical transmittance exceeding 90 % at 0°–60° incidence angles within optical ranges of 1.42, 1.7, and 3–5 μm. Compared to a zinc sulfide (ZnS) window with a UHMA on its surface, the THM improved the average transmission by 34.3 % over wide angles while allowing microwave scattering control. Broadband polarization-independent, low-crosstalk imaging, and hydrophobic characteristics were demonstrated. This study provides a design approach for multifunctional devices with synergistic optical and microwave regulation, particularly for optical transparency in microwave devices.

## 1 Introduction

The development of cross-band multifunctional electromagnetic wave-compatible control enables integration of multiple fields. Novel optoelectronic integrated materials and devices have gained significant attention for their applications in electromagnetic control camouflage [1], [2], [3], [4], remote sensing communication [5], [6], [7], imaging [8], [9], [10], [11], and ensemble algorithms [12], [13], [14]. Integration of optoelectronic detection systems across different modalities and spectral ranges with electromagnetic modulation materials has revealed limitations of traditional transparent electromagnetic camouflage window materials [15]. These include narrow optical transmission bandwidths, substantial transmission losses in the infrared range, and significant optical dispersion in electromagnetic modulation unit structures, which restrict the applications of multifunctional integrated devices, particularly in optical windows for airborne pods, guidance modules, and communication equipment [16], [17]. Therefore, exploring low-crosstalk and efficient cross-spectrally compatible modulation technologies is imperative [18].

Multiband compatible control technology has become a research hotspot [19], [20], [21], [22], [23]. However, compatible cross-scale modulation of visible light, lasers, infrared light, and microwaves remains limited due to design and fabrication challenges. Integrating optical transmission enhancement and microwave scattering control requires compatibility between transmission and reflection across spectral ranges [24], [25], [26]. The core challenge for multiband compatibility arises from electromagnetic scale effects: a single structure undergoes sharp transitions between Rayleigh scattering (*d*/*λ* ≪ 1), Mie resonance (*d*/*λ* ≈ 1), and geometric optics regimes (*d*/*λ* ≫ 1) across operational frequencies, where *d* denotes the characteristic geometric dimension and *λ* represents the incident wavelength [27], [28]. Complex decoupling modulation is needed at interfaces to satisfy conflicting parameters and eliminate resonance interference. Multiscale hierarchical metasurfaces offer an effective approach [29]. Zhang et al. developed a sandwich structure with a cross-ring-shaped resonator and reflective backplane for visible-NIR transparency and microwave absorption [30]. Ge et al. created a visible-light-transparent broadband microwave absorber using double-layer indium-tin oxide (ITO), achieving 90 % absorption in 24.49–34.39 GHz (forward) and 25.26–34.28 GHz (backward) bands respectively [31]. Qu et al. developed a visible-light transparent broadband microwave absorber with infrared camouflage functionality, attaining >90 % absorption across 7–23 GHz, thereby advancing compatible regulation of infrared stealth and microwave absorption [32]. While these studies achieved optical-microwave-compatible regulation, they solely rely on absorption mechanisms for microwave camouflage, neglecting to explore alternative approaches and failing to consider efficient optical detection during the electromagnetic camouflage process. As an optical camouflage window, the THM is required to not only maintain excellent optical performance but also possess stable camouflage capabilities, which are not limited to microwave and infrared camouflage. It is well-known that microwave absorption mechanisms rely on material losses to convert absorbed electromagnetic waves into thermal energy, which undoubtedly increases the risk of exposure through thermal signals – this contradicts the design concept of camouflage in the present work. Additionally, microwave absorption mechanisms may suffer from severe performance degradation under oblique incidence, and existing studies have not discussed their angular dependence [33], [34]. In contrast, beam manipulation achieves camouflage by controlling the phase and amplitude of electromagnetic waves through geometric structures to scatter them into non-threatening directions, exhibiting favorable angular insensitivity. Most described transparent-compatible modulation devices are limited to the visible spectrum [35], [36], [37] and suffer from impedance mismatch under large-angle electromagnetic wave incidence, leading to degradation of transverse magnetic (TM) or transverse electric (TE) transmission efficiency. Simple layered metasurfaces cause interference losses in optical detect imaging with small bandwidth for large-angle optical transmission and high-order optical diffraction losses. Therefore, when designing cross-band compatible control window devices for multimode fusion detection systems, the following aspects need to be considered: (i) enhancing multispectral optical transmission for multitarget detection and (ii) reducing broadband radar cross section (RCS) while maintaining low optical diffraction loss characteristics for electromagnetic camouflage. Additionally, a stable, high-precision, large-area manufacturing process is required to achieve trans-scale fabrication optical transmittance.

This paper introduces a trans-scale hierarchical metasurface (THM) optical window for wide-angle optical transmission enhancement and electromagnetic scattering regulation to address multiband compatibility and interference in dual-band lasers, infrared, and microwaves. The THM includes a transmission-enhanced micro-nano cone array (TMCA) and an ultrafine hollow metal array (UHMA), integrated through a multistage nanolithography process. Constructed with a four-layer architecture on a multispectral zinc sulfide (ZnS MS) substrate, simulation analyses and experimental results show the THM has high transmission in laser and infrared bands (average transmittance >90 % at 1.42, 1.7, and 3–5 μm for incidence angles from 0° to 60°), and efficient RCS reduction in the microwave band (≤−10 dB at 9.5–17.5 GHz). Ultra-low normalized high-order diffraction intensity (0.002965 %) and polarization-independent, hydrophobic (above 110°) characteristics were also shown. To the best of our knowledge, this is the first reported composite window device with RCS reduction and optical transmission enhancement. The design model and scheme offer advantages in functional enhancement, bandwidth expansion, structural integration, device miniaturization, and application diversification, showing potential for precision optoelectronic systems in aerospace, guided detection, and communication window devices.

## 2 Design and simulations

### 2.1 Design concept of THM

**Figure 1:**
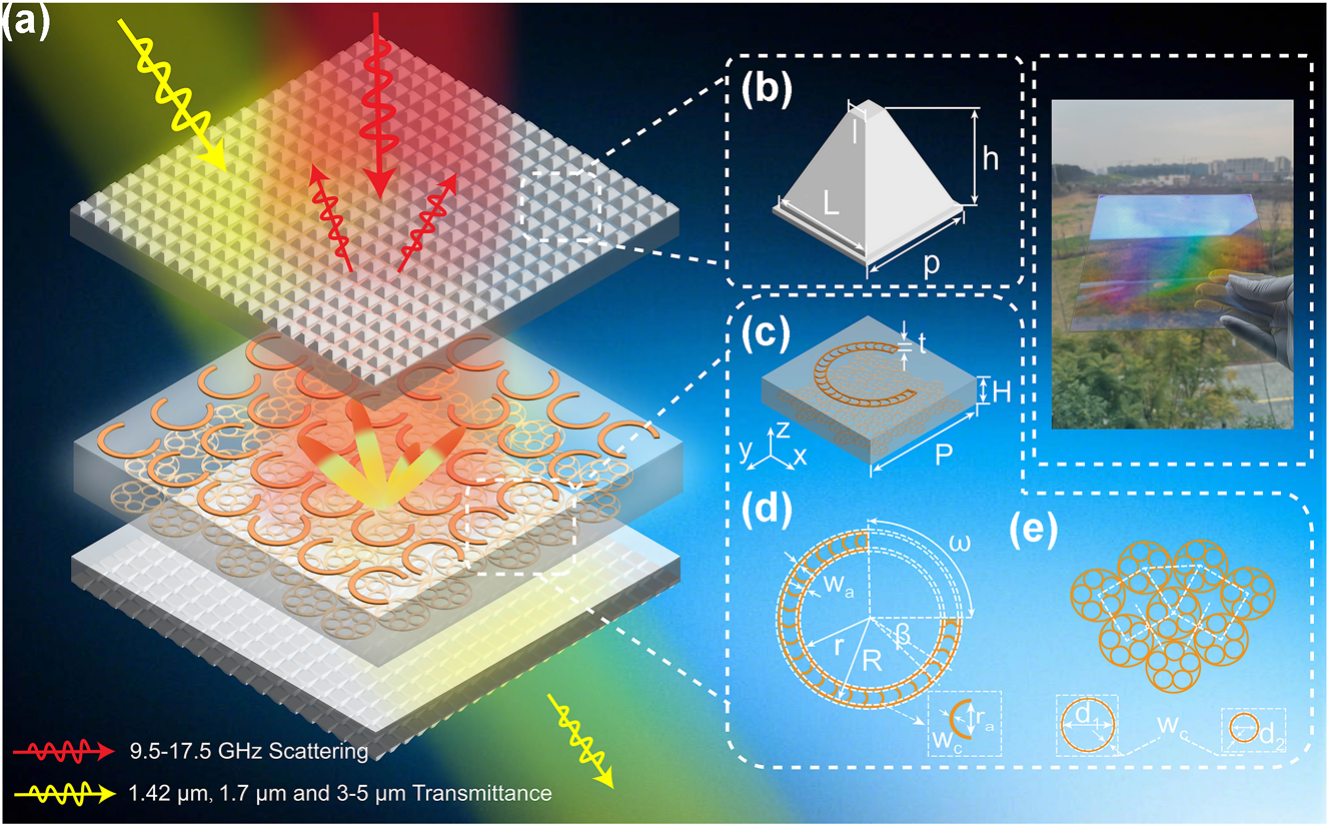
Schematic diagram of an optically transparent, multifunctionally compatible metasurface. (a) Functional schematic of different frequency bands. When multispectral electromagnetic waves incident on the metasurface include a dual-band laser at wavelengths of 1.42 and 1.7 μm, as well as mid-infrared light in the 3–5 μm range, both the laser and infrared light pass directly through the metasurface, exhibiting significant angular insensitivity. In contrast, the microwave components are guided in a non-specular direction by the metasurface, enabling compatibility in the camouflage of microwave, infrared, and visible light. (b) Schematic diagram of TMCA. (c) Schematic of the microwave unit structure. (d), (e) Schematics of the superstructure and substructure of the unit structure, respectively.

Figure 1(a) shows a schematic of the proposed THM. A ZnS MS was employed as the base layer connecting the bilayer metasurface structure to meet multispectral front-end device requirements. ZnS MS was chosen for its transparency, low absorption loss, stability, density, processing ease, and environmental adaptability. The topmost and bottom layers consisted of a bilayer with periodic TMCA arrangement made from materials matching the substrate (Figure 1(b)). TMCA serves two functions in the THM device. First, it modulates the dual-band laser at 1.42 and 1.7 μm while enhancing infrared band transmission (3–5 μm). The TMCA regulates optical scattering and guided-mode resonance (GMR) by controlling the material’s equivalent refractive index, reducing large-angle transmitted energy loss. Furthermore, independent manipulation of GMR effects via TMCA structural parameter modulation enables significant wide-angle optical transmission enhancement at dual-band laser positions. Second, given the orders-of-magnitude scale disparity between the TMCA and UHMA architectures, the TMCA behaves as an ultrathin effective monolayer with a homogeneous refractive index in the microwave regime, consequently exhibiting negligible perturbations to the electromagnetic scattering modulation. As illustrated in Figure 1(a), when electromagnetic waves impinge on THM, the microwave components penetrate the upper TMCA layer and undergo reflection in the precisely engineered UHMA. Through phase modulation by UHMA, the reflected waves exhibit an RCS reduction in the specular direction, whereas laser-infrared waves at designated bands experience transmission enhancement through the TMCA, ultimately penetrating the THM. This physical process constitutes an essential mechanism for achieving a multifunctionally compatible cross-scale hierarchical modulation. Regarding the principles of dielectric permittivity engineering for enhancing optical transmittance in thin films, achieving cross-band wide-angle transmission enhancement requires a graded-index (GRIN) or periodic multilayer structures with alternating high/low refractive indices. However, the resulting increased thickness and spatially dispersive effective permittivity may induce microwave phase errors or parasitic surface waves, thereby complicating scattering modulation and device design. TMCA offers a robust solution for tuning multifunctional compatibility, significantly enhancing the overall performance stability of the device. This is achieved by ensuring that the structural material is consistent with the substrate material, thereby mitigating issues related to the dielectric mismatch that could lead to delamination of the multilayer structure.

As depicted in Figure 1(c), UHMA comprises a symmetric cubic phase-distributed upper chord-lunar metallic metasurface array and a lower densely interconnected multiloop nested metallic metasurface array. UHMA fulfills three critical roles. First, the interferometric phase cancellation of reflected waves generated by the upper metallic metasurface structure effectively reduces echo signals in the specular direction, achieving efficient RCS reduction within the broadband microwave range of 9.5–17.5 GHz. Second, the low-duty cycle skeletonized metal ring design facilitates visible-light transparency. Finally, compared to traditional optically transparent metal mesh grid structures, the matching upper- and lower-ring configurations reduce the effects of normalized high-order diffraction intensity and minimizing interference in optical imaging detection, thereby broadening the application scope of the device. Furthermore, as the overall structural unit phase exhibits a quarter-geometrically symmetric distribution, it demonstrates near insensitivity to the polarization state of the incident waves across both the optical and microwave bands.

### 2.2 Design and simulation of UHMA

Based on the proposed cross-scale hierarchical modulation concept and perspective of multifunctional compatibility design, the initial focus is on developing a low-diffraction, broadband, and high-efficiency RCS curtailment structure that exhibits robust compatibility modulation functionality. There are two common methods to reduce the return signal. First, a thin film of wave-absorbing material is applied to the surface of the device to shape the target waveform. Second, the metasurface modulates the electromagnetic wave, redirecting it to a non-threatening angle. Notably, wave-absorbing materials are typically only effective within a specific frequency range. Increasing the thickness of the film layer may be necessary to enhance wave absorption. However, a critical consideration is that these materials also generate thermal radiation during operation, which contributes to increased background noise in the optical infrared spectral band, potentially causing issues in the detection window. Inspired by the principle of coded metamaterials [38], this study designed a hollow metal-metasurface unit structure (Figure 1(c)) based on geometric phase modulation. By constructing different opening directions to adjust the reflection phase, the phase distribution of the array was further formed, thereby achieving RCS reduction across a wide bandwidth. Moreover, the simulation aimed at verifying the cross-spectral modulation of multifunctional devices encounters challenges because of the nearly 10^4^ orders of magnitude difference between the microwave frequency band and the optical spectral band of the proposed structure. Each unit cell of the UHMA corresponds to more than 20 million TMCA, presenting significant barriers to cross-scale simulation analysis. Currently, commercial software is struggling to conduct accurate large-scale cross-scale analyses. Many common cross-scale analyses tend to overlook the impact of microscale structures on the cross-band performance, which may be acceptable for energy amplitude modulation.

**Figure 2:**
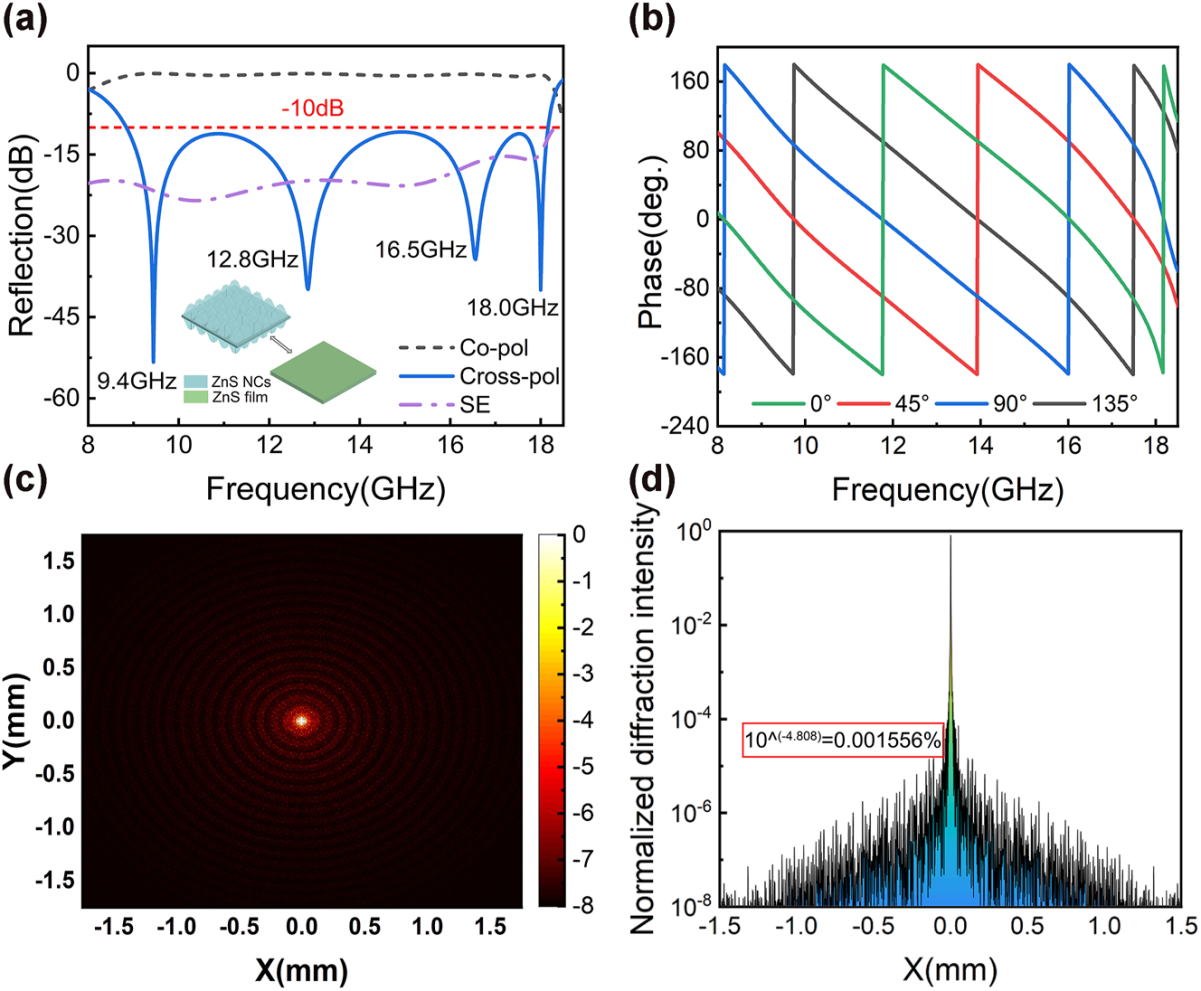
Simulated results of (a, b) unit microwave interactions and (c, d) diffraction patterns. (a) Simulation results for co-polarization and cross-polarization of the unit structure, with the inset illustrating the simulation using TMCA equivalent to a ZnS MS planar film layer. (b) Phase changes observed at different angles of structural rotation. (c, d) Diffraction pattern of the metallic structure and the normalized diffraction intensity distribution, respectively.

In this study, a combination of theoretical equivalent calculations and simulations was adopted to analyze the cross-scale interactions of UHMA for microwave modulation and TMCA for optical modulation. Based on the above method, the RCS reduction performance of the THM was first analyzed. The simulation of the amplitude and phase of the UMWA unit structure is shown in Figure 2. In this analysis, TMCA was treated as a homogeneous medium following the equivalent medium theory [39], as shown in the inset of Figure 2(a), the upper and lower TMCA layers were equivalently modeled as homogeneous dielectric material layers in a simulation of the UHMA unit structure. The equivalent dielectric simulation of the periodically arranged 2D TMCA is provided in Section S1 (Supporting Information), where the structural period and bottom length are 1.44 and 1.21 μm, respectively, the duty cycle is 84 % (length/period), and the structural depth is 1.54 μm. For the microwave frequency band simulation, when the equivalent thickness of the TMCA film layer is 1.54 μm, its equivalent refractive index is 2.2405, which is smaller than the refractive index of ZnS, 2.45, thus satisfying the equivalent medium theory (Supplementary Materials S1). Broadband microwave scattering control is crucial to render the radar detection of microwave signals ineffective. An efficient broadband RCS reduction control structure was proposed consisting of a top-layer crescent-shaped metallic metasurface array unit made of copper with a thickness of 100 nm, as shown in Figure 1(d). The specific structural parameters are as follows: dielectric period of *P* = 7 mm, thickness of *d* = 2.5 mm, outer ring line width of *w*_*a*_ = 10 μm, inner hollow structure semicircular line width of *w*_*c*_ = 5 μm, radius of *r*_*a*_ = 45 μm, angle between semi-circular ring is *β* = 2.5°, outer ring inner diameter size is *R* = 2.68 mm, inner ring inner diameter size is *r* = 2.58 mm, and opening angle is *ω* = 52°. The lower densely connected multiring nested metallic metasurface array, with the same material and thickness as the upper array, as shown in Figure 1(e), consists of a large circle nested with five small circles, which are internally tangent to each other, and all of them are very narrow metallic thin wires with a linewidth of *w*_*c*_ = 5 μm, in which the inner diameter of the large circle is *d*_1_ = 1,070 μm and that of the inner small circle is *d*_2_ = 386 μm. The top-layer crescent-shaped geometric phase unit is illuminated by right- or left-handed circularly polarized waves. The incident beams with varying polarization states underwent polarization conversion within the frequency range of 8.9–18.2 GHz. When circularly polarized light (CP) was vertically incident on the unit cell surface, Figure 2(a) shows the simulated reflection coefficient of the unit structure. Notably, the cross-polarized reflection amplitude is significantly reduced by more than −10 dB within 8.9–18.2 GHz. Additionally, the unit cell exhibits four resonance frequencies at 9.4, 12.8, 16.5 GHz, and 18.0 GHz within the desired operating frequency range. Simultaneously, strong copolarization reflection was noticed at 8.9–18.2 GHz. To further investigate the geometric phase modulation characteristics of the UHMA unit within the operational frequency range, Figure 2(b) shows the reflection phase characteristics corresponding to different rotation angles of the crescent-shaped metasurface unit under the normal incidence of left-handed circularly polarized waves. The simulation results indicate that changes in the rotation angle have a relatively small impact on the reflection amplitude (Supplementary Material S2, Figure S1(a)). As the rotation angle increases in increments of 45°, a modulation range of −*π* to *π* is achieved, accompanied by a nearly parallel phase response, resulting in destructive interference among the reflected waves from these units. Additionally, we simulated the effects of oblique incidence on the unit structure for both co-polarization and cross-polarization (Supplementary Material S2, Figure S1(b)). The results indicate that copolarization remained unaffected, whereas cross-polarization exhibited only slight fluctuations. It is anticipated that the structure will demonstrate an effective RCS reduction within a 30° range of oblique incidence, although there may be a slight narrowing of the bandwidth. In the initial design phase, this study also considered the optical transmission loss of the dual-layer ultrafine metallic line structure and its low crosstalk in infrared imaging. Simulations and testing revealed that the overall loss of the metallic structure was approximately 7 % (Supplementary Material S3). To mitigate high-order diffraction intensity, a bottom circular reflective layer was designed with large outer circles interconnected sequentially by equilateral triangles and squares. The smaller inner circles are tangential to each other and are inscribed within the larger circles, resulting in a more densely packed and unordered arrangement. Figure 2(c) and (d) show the diffraction patterns of the metallic structure and the normalized high-order diffraction intensity, respectively. These results were obtained through matrix programming calculations using software. The simulation indicates that the maximum normalized high-order diffraction intensity is approximately 0.001557 %.

### 2.3 Microwave full-wave simulation

Compared with the traditional checkerboard arrangement, which redirects scattered waves to the four azimuthal angles of 45°, 135°, 225°, and 315°, we adopted a symmetric cubic phase arrangement. This approach effectively minimizes the strong scattering side lobes at these angles, while promoting the scattering of echoes. Unlike algorithmic optimization methods, which seek the optimal arrangement through exhaustive searches, this technique is more intuitive and calculates the phase mask for each unit using the cubic-phase formula [40]:

(1) $$\varphi \left(x_{c},y_{c}\right)=\frac{\alpha }{D^{3}}\left(\left|x_{c}^{3}|+|y_{c}^{3}\right|\right)$$

where *φ*(*x*_*c*_, *y*_*c*_) is the phase mask of the unit structure corresponding to the position on the metasurface, *α* is the modulation index, and *x*_*c*_, *y*_*c*_ are the coordinates of the metasurface unit cell on the *xoy* plane, with *D* being the side length of the metasurface.

**Figure 3:**
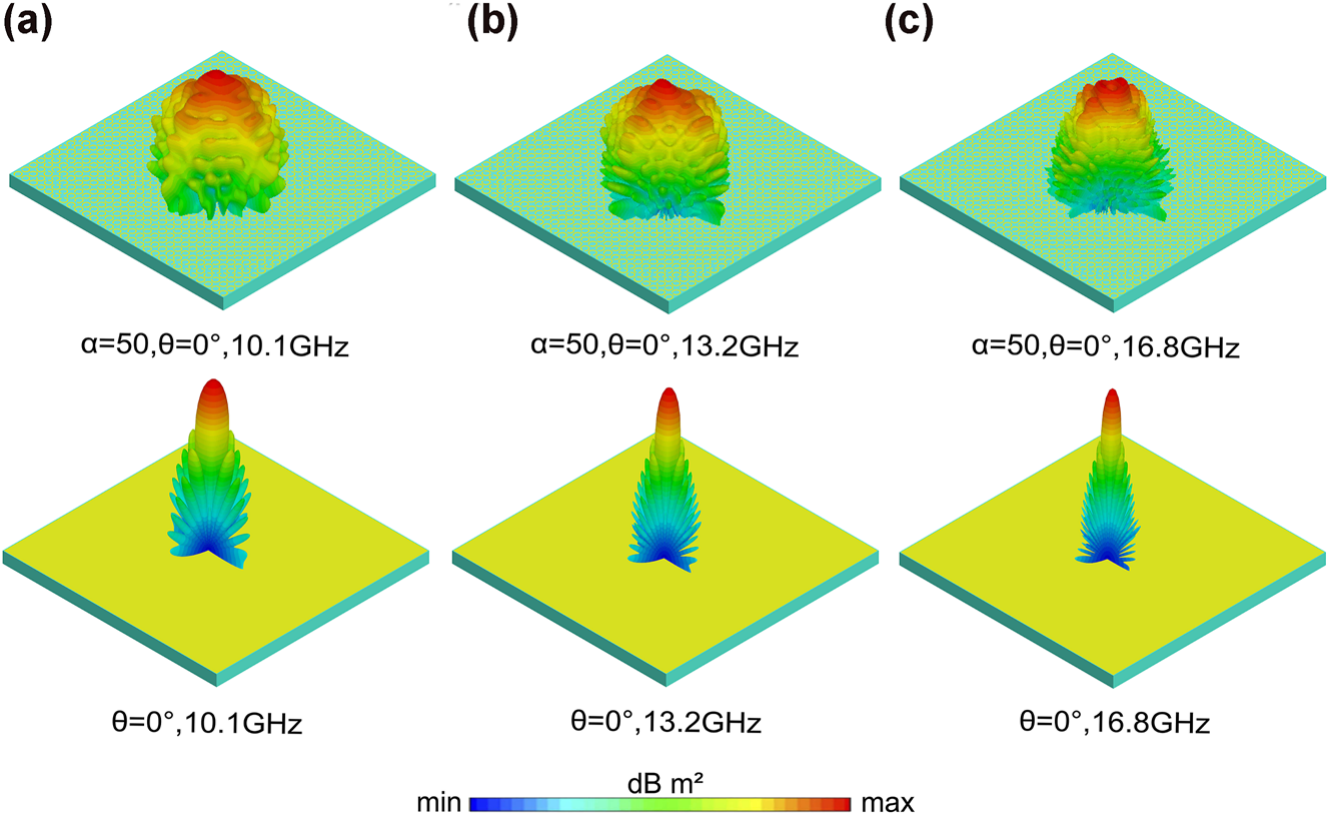
Simulated far-field 3D scattering patterns of the metasurface and the metal plate. Panels (a–c) present the 3D far-field scattering patterns of the metasurface and the metal plate under normal incidence (*θ* = 0°) at frequencies of 10.1, 13.2, and 16.8 GHz, respectively.

To investigate the influence of the modulation index *α* on the reduction in the RCS of the metasurface, for the impact of *α* values of 25, 50, 75, and 100, on the RCS of the same metasurface were analyzed via simulations (Supplementary Material S4, Figure S3). In this study, a metasurface consisting of 28 × 28 unit cells with dimensions of 200 × 200 mm^2^ was fabricated to determine that the optimal modulation index was 50 based on our simulation results. A full-wave simulation was performed to quantitatively characterize the metasurface scattering properties. Figure 3(a)–(c) illustrate the far-field 3D scattering patterns of both the metasurface and metal plate at frequencies of 10.1, 13.2, and 16.8 GHz, respectively. The backscattered energy from the cubic-phase metasurface was diffused and redirected in all directions with no prominent scattering side lobes. In contrast, for a perfect electric conductor (PEC) plate with the same dimensions, reflection predominantly occurs in the mirror direction, as dictated by Snell’s law, leading to strong scattering lobes along the line of sight. To align our findings with practical scenarios, we also simulated the effect of the oblique incidence of electromagnetic waves on the metasurface (Supplementary Material S4, Figure S4). These results demonstrate that the metasurface maintains effective backscattering suppression at an oblique incidence of 30°. However, as the incidence angle increased, the bandwidth of the suppression effect gradually decreased, resulting in a diminished performance. This degradation is primarily attributed to the phase distortion under oblique incidence, suggesting that the metasurface is better suited for complex real-world conditions. To validate the optical performance of the TMCA, the UHMA can be treated as a transmissive loss layer in equivalent simulations. In applications where these devices serve as windows, light beams passing through the metal layer experience transmissive losses, even at very low thicknesses. This effect is particularly pronounced for large-angle incidences and within the infrared spectrum. To mitigate this issue, both double-layer UHMA designs incorporate ultrafine metallic rings, which significantly enhance the fundamental transmissivity in the optical range.

### 2.4 Design and simulation of TMCA

To investigate the changes in the transmittance of the ZnS MS under varying conditions, simulations were conducted using FDTD Solutions software. Simulations were designed for bare ZnS MS, ZnS MS with metallic structure, and ZnS MS with an added TMCA layer. The focus is on the transmittance variation trend after introducing the metal layer and TMCA in the simulation, the introduction of a metallic structure to the substrate surface results in a decrease in transmittance. Conversely, the application of a TMCA layer significantly enhanced the overall transmittance, suggesting that the design could partially mitigate the loss caused by the metallic structure (Supplementary Material S5). The period of the designed UHMA is significantly larger than the incident light wavelength, enabling the use of the porosity concept of scalar diffraction theory to characterize the optical transmissivity of the material. An equivalent simulation indicated that the optical transmissivity loss associated with the UHMA could be reduced by approximately 7 %. To enhance the performance of optical imaging windows and broaden their applications, it is essential to structurally regulate optical material systems. Inspired by insect compound eye biomimetic structures with capabilities for optical field capture and control [41], [42], [43], in the design process, a visible-light transparent TMCA structure with dual-frequency laser-infrared compatibility was developed, exhibiting wideband and wide-angle transmission enhancement characteristics (illustrated in Figure 1(b)). When the subwavelength structure is firmly bonded to the substrate, the high refractive index and high optical mode density of the ZnS material create favorable conditions for the interaction between the Mie resonance scatterers and light. Enhanced Mie scattering makes it easier for the light generated by the resonance to couple with the substrate, resulting in strong forward scattering, effectively guiding the incident light to the substrate and enhancing the transmittance [44], [45]. To eliminate the resonance effects, the objective was to induce a redshift in the reflection spectrum, thereby regulating the GMR within the target working spectrum and broadening the transmission spectral range for oblique incidence. Furthermore, by implementing an underlying array structure to guide the direction of outgoing electromagnetic waves, echo reflections from lower-material interfaces can be reduced by minimizing the internal reflection losses caused by abrupt changes in material properties, such as the refractive index, thickness, and density. The shape, period, and duty cycle of micro-nanostructures are the key parameters governing the antireflective (AR) effect. Given that the AR performance of microstructures is highly sensitive to these factors, geometric modifications can alter the volume fraction at the optical interface, resulting in changes to the fundamental properties of the materials (such as the refractive index, conductivity, and permeability), which in turn affect the transmission characteristics of the device. Preliminary research indicates that conical morphologies exhibit superior optical antireflective characteristics compared with porous morphologies [46], [47]. Therefore, a TMCA was designed that was aligned with the ZnS MS material system of the substrate, avoiding abrupt refractive index changes associated with multilayer film materials, thus reducing internal reflection within the structure.

**Figure 4:**
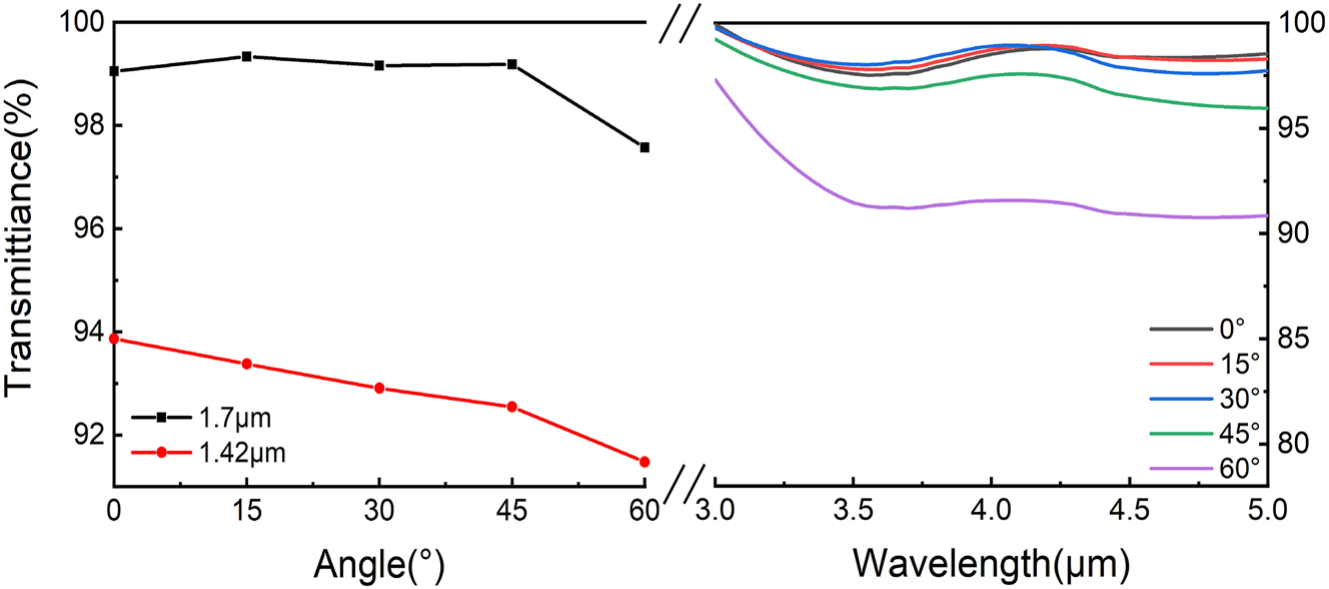
The transmittance of the optimized TMCA structure for dual-frequency lasers and the 3–5 μm infrared band.

To ensure consistency in the structural fabrication, this study optimized and controlled the parameters of both the upper and lower unit structures. A schematic of the structural dimensions is shown in Figure 1(b). The amplitudes for the dual-frequency laser and infrared band under plane-wave irradiation were calculated to characterize the optical transmittance of the microstructure. The results, illustrated in Figure 4, demonstrate that the TMCA structural unit achieves an average transmittance of >90 % in the 1.42, 1.7, and 3–5 μm wavelength ranges for incident angles from 0° to 60°. Theoretical predictions for the proposed dual-layer TMCA structure can be made using the two-dimensional subwavelength medium spatial optical wave vector diffraction transmission formula:

(2) $$k_{0}n_{i}\sin\theta_{in}\pm m\frac{2\pi }{P_{x,y}}=k_{0}n_{\text{eff}}$$

where *k*_0_, *n*_*i*_, *θ*_in_, and *m* are the free space wave vector, the refractive index of the incident medium, the angle of the incident light, and the diffraction orders in the *x* and *y* directions, respectively. *P*_*x*,*y*_ represents the periodic sizes along the *x*- and *y*-axes of the unit cell. Based on the aforementioned formula, the periods in the *x* and *y* directions can be calculated theoretically. In the design of the TMCA structure, both the effective refractive index and period of the structure can be adjusted to control the spatial wave vector transmission characteristics of electromagnetic waves. This adjustment leads to a red shift in the resonance wavelength, which contributes to reflective losses. Using equation (2), the structural periods within the 3–5 μm wavelength range could be generated, thereby enabling only the zeroth-order diffraction wave to propagate, while higher-order diffraction waves remain as evanescent waves. Furthermore, we introduce the constraint coefficient *δ* = (*φ*_1_ + *φ*_2_ − 2*k*_0_ *d*_*i*_)/2 for the reflection phase, where *φ*_1_ and *φ*_2_ are the structural reflection phases, *d*_*i*_ refers to the structural phase thickness (specifically for 1.42 and 1.7 μm). The period of the designed TMCA structure can be calculated using the following formula:

(3) $$P_{x,y}=\frac{\lambda \sin^{2}\delta }{\max\left(n_{\text{eff}},n_{i}\sin\theta_{in}\right)+n_{i}\sin\theta_{in}}$$

where *λ* is the wavelength, *n*_eff_ is the effective refractive index of the microstructure, *n*_*i*_ is the refractive index of air, and *θ*_in_ is the angle of incidence. In our design, the structural periods for 1.42, 1.7, and 3–5 μm are iteratively calculated using formula (3). Theoretical calculations and simulations are performed assuming the light is incident vertically onto the structure’s surface, with *θ*_in_ = 0°. After phase calculations, a value of 0.7 is taken, resulting in a period of 1.44 μm to achieve higher transmittance in the infrared broadband and dual-band laser. From (3), it can be observed that the diffraction suppression capability of the unit structure strongly depends on the joint modulation of *n*_eff_ and *P*_*x*,*y*_. The height of the micro-nanostructure and *n*_eff_ can establish an equivalent approximate numerical relationship using:

(4) $$H\geq \frac{\pi }{2k_{0}\sqrt{\max\left(n_{\text{eff}},n_{i}\right)\cdot n_{i}}}$$

**Figure 5:**
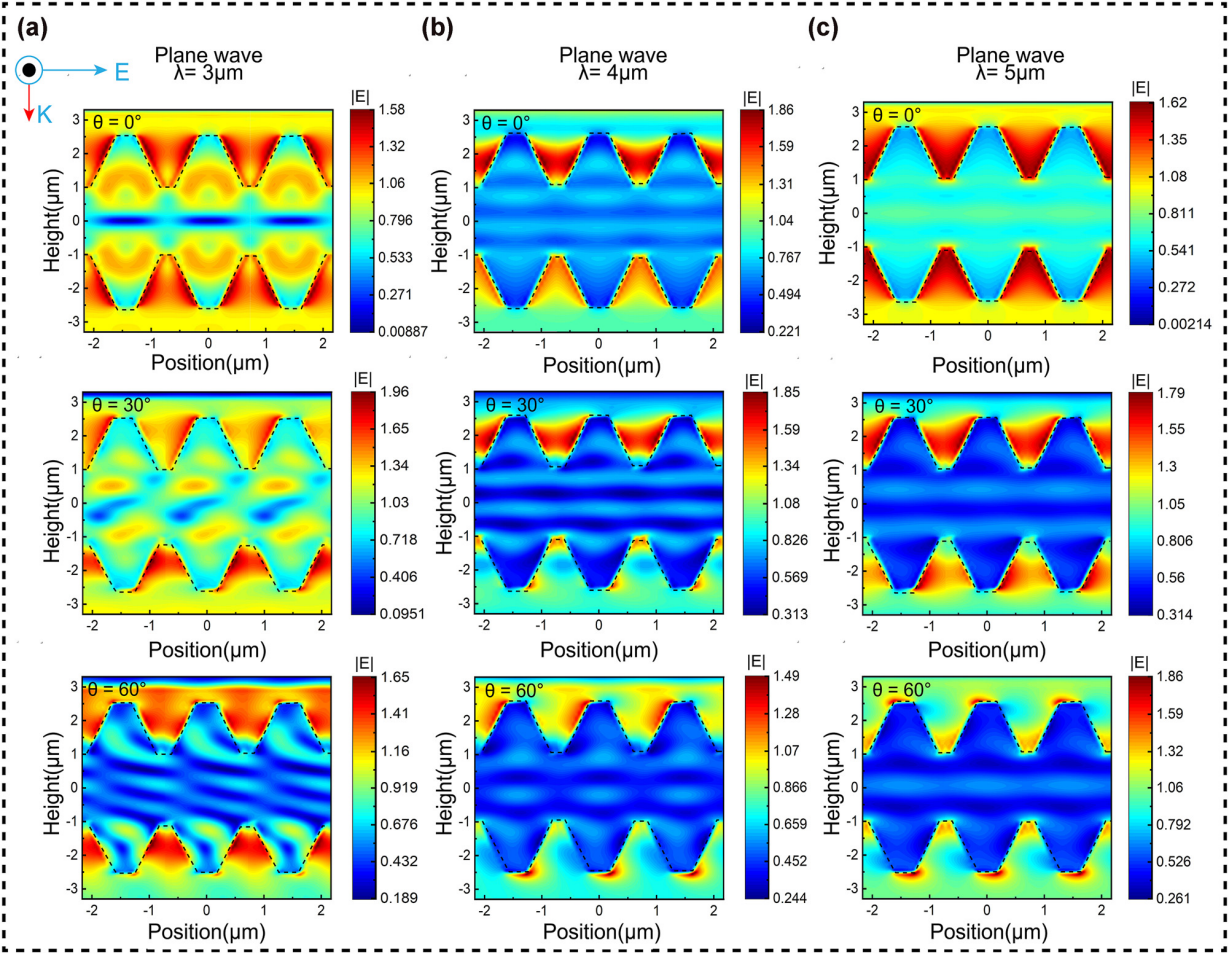
Simulation validation of the electric field distribution is conducted. (a–c) Electric field distribution of the TMCA structure at 3 μm, 4 μm, and 5 μm wavelengths under 0–60° incidence.

After calculations, the designed unit structure height is set at 1.54 μm, and the other structural parameters of the unit are *L* = 1.21 μm and *l* = 0.34 μm. To validate the theoretical formula and analyze the energy transmission mechanism of the interaction between incident electromagnetic waves and the TMCA structure, simulations of the electric field were conducted. Figure 5 describes the distribution of the plane electric field intensity under vertical and inclined incidence conditions in the 3–5 μm range. A TMCA structure unit with a period of 1.44 μm and a height of 1.54 μm was selected to accurately represent the electric field distribution. Parameter optimization of the conical structure was performed using a simulation design. Analysis of the electric field distribution revealed that Mie scattering in subwavelength structures significantly amplifies localized fields within interstitial regions, thereby modulating optical transmission [44], [47]. By designing suitable subwavelength structures, it is possible to manipulate this resonance effect to adjust the light transmittance [48]. When the incident wavelength falls within the 3–5 μm range, it is larger than the structural dimensions, leading to considerable local field enhancement between structures, effectively confining incoming light. While diffraction effects increase with the angle of incidence, a substantial local field enhancement remains among the structures, facilitating strong transmission even at larger angles. To investigate the transmission performance at wavelengths of 1.42 and 1.7 μm, we studied the electric field distributions at these wavelengths (Supplementary Material S6). In addition, we conducted an in-depth discussion on the impact of different structural heights and widths on the transmittance of the structure (Supplementary Material S7).

### 2.5 Simulation details

The electromagnetic characteristics of the UHMA unit cell, including amplitude and phase responses, as well as the RCS reduction performance of the metasurface, were numerically investigated using the commercial software CST Microwave Studio. Initially, the frequency-domain solver was employed to characterize the reflection amplitude and phase variations of the unit cell structure. The unit cell boundary was imposed along both *x*- and *y*-directions, and the open boundary was implemented in the *z*-direction. Subsequently, the time-domain solver was utilized to obtain 3D far-field scattering patterns of the UHMA. The open boundary was applied in all three spatial dimensions (*x*, *y*, and *z*), with excitation provided by a plane wave propagating along the negative *z*-axis. The relative permittivity of the ZnS MS was experimentally determined through laboratory measurements using standard characterization techniques. The commercial software FDTD Solutions was employed to numerically analyze the transmittance characteristics of the subwavelength optical antireflection structures. Under normal incidence conditions, the periodic boundary was applied along both *x*- and *y*-axes, with perfectly matched layers (PMLs) implemented along the *z*-direction. For broadband oblique incidence simulations, the broadband fixed-angle source technique (BFAST) was employed for excitation source, with the corresponding BFAST boundary applied in the *x*- and *y*-directions and PMLs in the *z*-direction. When evaluating transmittance at dual laser wavelengths under oblique incidence, the simulation boundary conditions were adjusted to the Bloch type, and the source type was set to Bloch/periodic. Multiple field profile monitors were strategically positioned to record the spatial distributions of the electric field, and power monitors were implemented to obtained the reflectance and transmittance spectra. All the simulations were conducted with a consistent time window of 10,000 fs to ensure proper convergence of time-domain signals.

## 3 Experiments and results

**Figure 6:**
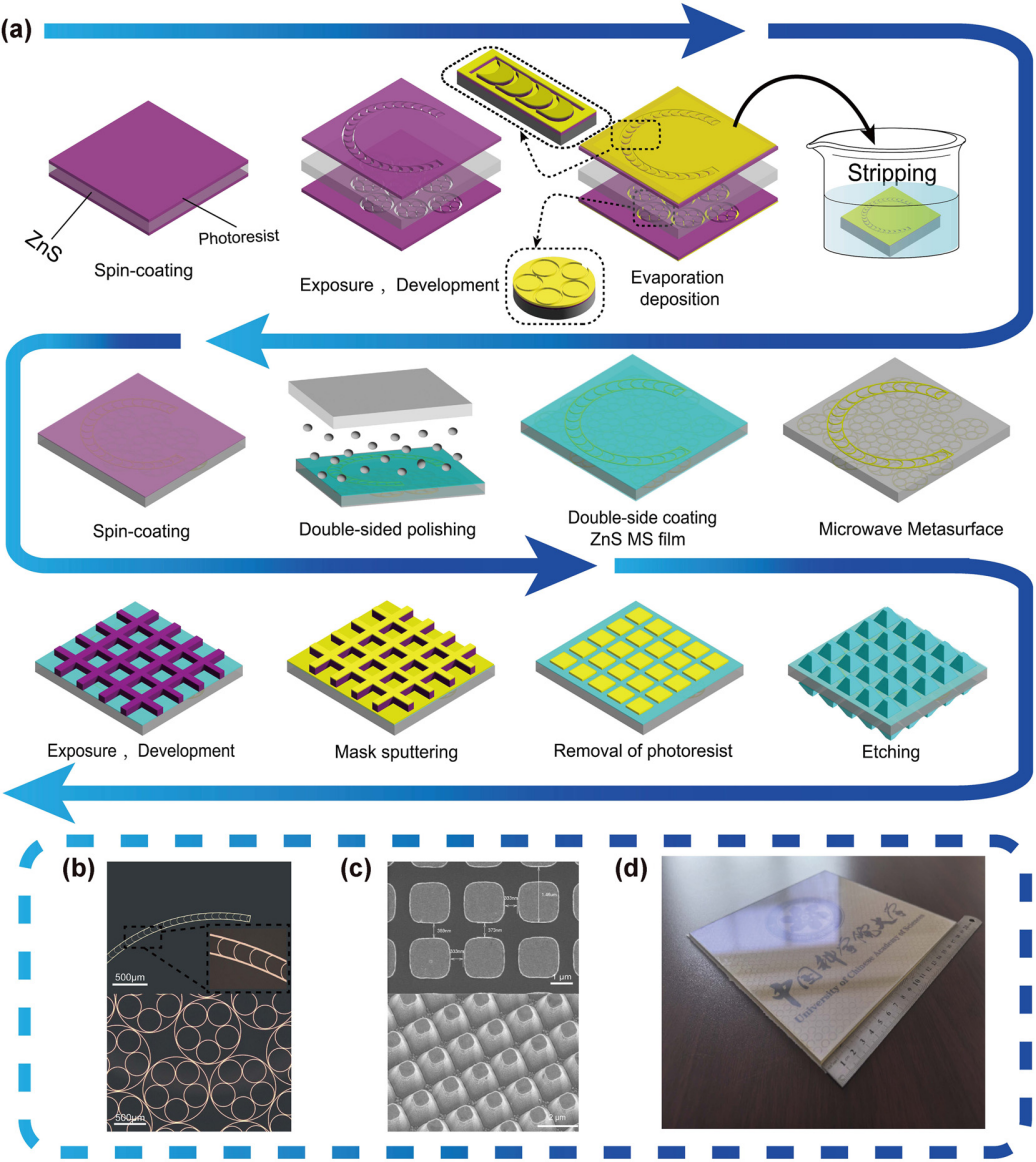
Fabrication and characterization of the THM device. (a) Schematic of the manufacturing process of UHMA and TMCA. (Top to bottom) Manufactured (b) physical image of the upper chord-lunar metallic phase-shifting structure of the microwave unit’s upper layer and the lower densely interconnected multiloop nested metallic reflective layer. (c) Metal mask layer of the subwavelength optical antireflection structure and the TMCA layer. (d) THM sample.

To experimentally verify the multispectral compatible regulation properties, a THM sample, measuring 200 × 200 mm^2^ with a minimum line width of 5 µm, was fabricated. Figure 6(a)–(c) show the processing flow, and the optical micro-scope and scanning electron microscope images of the UHMA and TMCA. Figure 6(d) shows the THM samples. The UHMA was first fabricated on both the front and back sides of the ZnS MS substrate using lift-off and evaporation processes. Then, ZnS MS thin-film layers were deposited on both sides of the substrate. The TMCA was fabricated using the sulfur hexafluoride (*SF*_6_) plasma reactive-ion-etching (RIE) process [23], [49]. This layer protects the metal structure from oxidation as well as regulates the interaction between the incident light and surface structure, thereby improving the transmission performance of the device (processing details provided in Supplementary Material S8). Future scalable deployment will utilize separately fabricated lithographic masters for high-throughput nanoimprint replication, establishing a cost-effective mass production pathway.

### 3.1 Microwave and optical performance characterization

**Figure 7:**
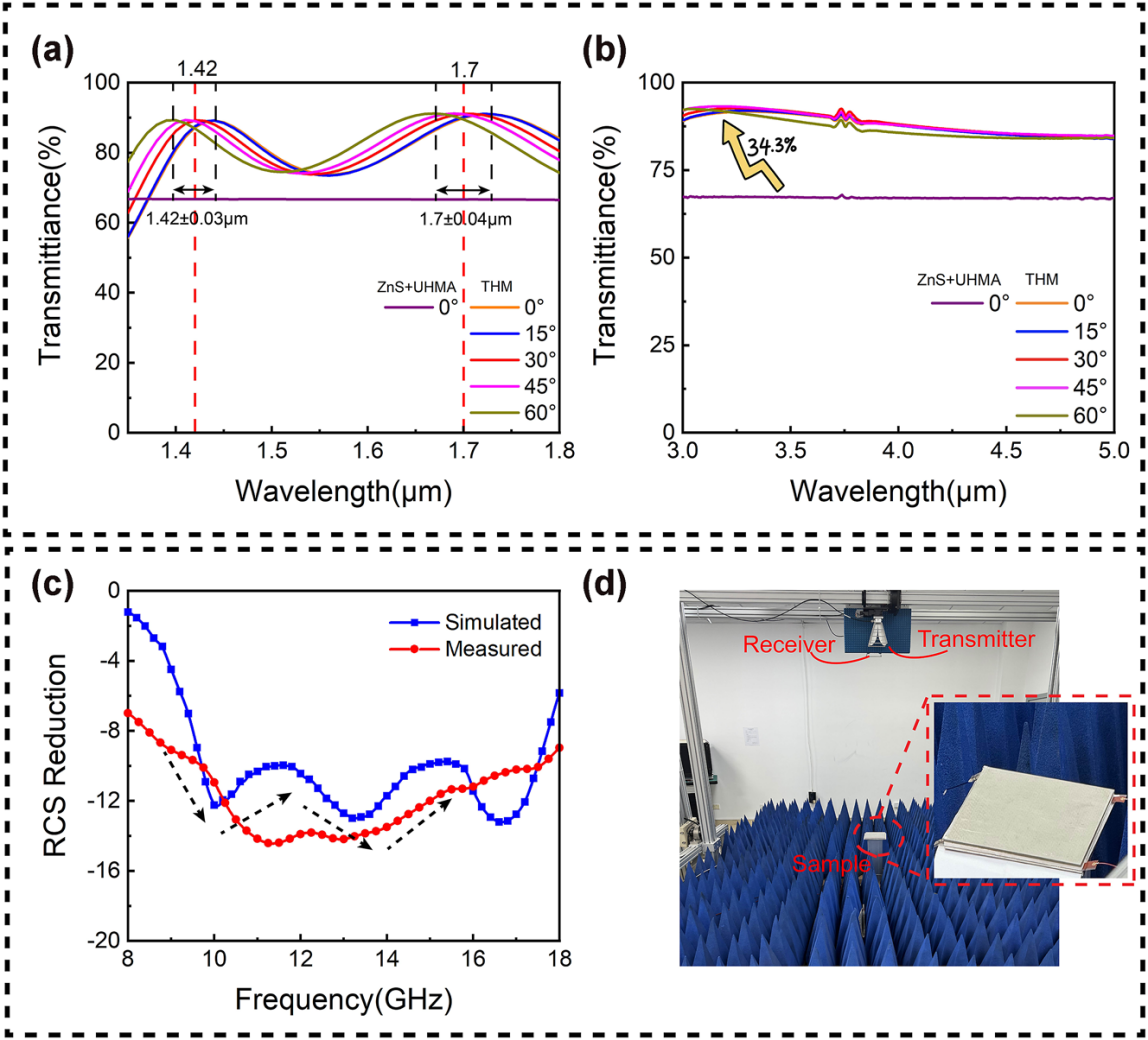
Testing of various performance parameters of THM. (a) Transmission testing of THM at 1.42 and 1.7 μm. (b) Transmission testing of THM in the 3–5 μm wavelength range with incident angles from 0° to 60°. (c) RCS reduction simulation and testing comparison of THM, with a frequency range of 8–18 GHz. (d) On-site testing environment demonstration.

The optical and microwave performances of the fabricated metasurfaces were characterized. Transmittance measurements were conducted in the near and mid-infrared ranges using a spectrophotometer (Lambda 1,050, PerkinElmer) and Fourier-transform infrared spectrometer (Vertex 80, Bruker). Figure 7(a) and (b) show that within the 0–60° wide-angle range, the average transmittance at 1.42, 1.7, and 3–5 μm is approximately 90 %, with angular response bandwidths of ±0.03 and ±0.04 μm at 1.42 and 1.7 μm, respectively. Compared with the ZnS MS that only underwent UHMA, the average transmittance of the THM has increased by approximately 34.3 % within the above frequency band. Notably, our transmittance curves were obtained for samples with microwave metal structures. The slight discrepancies between the measured and simulated results could be attributed to manufacturing defects and measurement errors. Next, we measured the microwave reflection characteristics of the THM using an arch measurement system, which was equipped with a vector network analyzer and two linearly polarized standard horn antennas (placed on an arch range) that functioned as the receiver and transmitter, respectively; the sample was located at its circle center (Figure 7(d)). A 200 × 200 mm^2^ metal plate served as a reference for calculating the RCS reduction, facilitating a clear assessment of the RCS reduction attributed to diffusion. Figure 7(c) compares the measured and simulated reflection performances. Evidently, our sample device achieves a −10 dB reflection level across a broad frequency range of 9.5–17.5 GHz. Notably, in the 10.8–14.2 GHz range, the reflection level drops significantly to approximately 14 dB. Owing to manufacturing tolerances, the experimental results show a slight different compared with the simulated results. However, the RCS showed an overall reduction, which was within an acceptable margin of error. In addition, the designed metasurface exhibited polarization independence, with almost identical the RCS reduction trends at TE- and TM-wave incidences (Supplementary Material S9).

### 3.2 Infrared thermography, diffraction, and hydrophobicity angle testing

**Figure 8:**
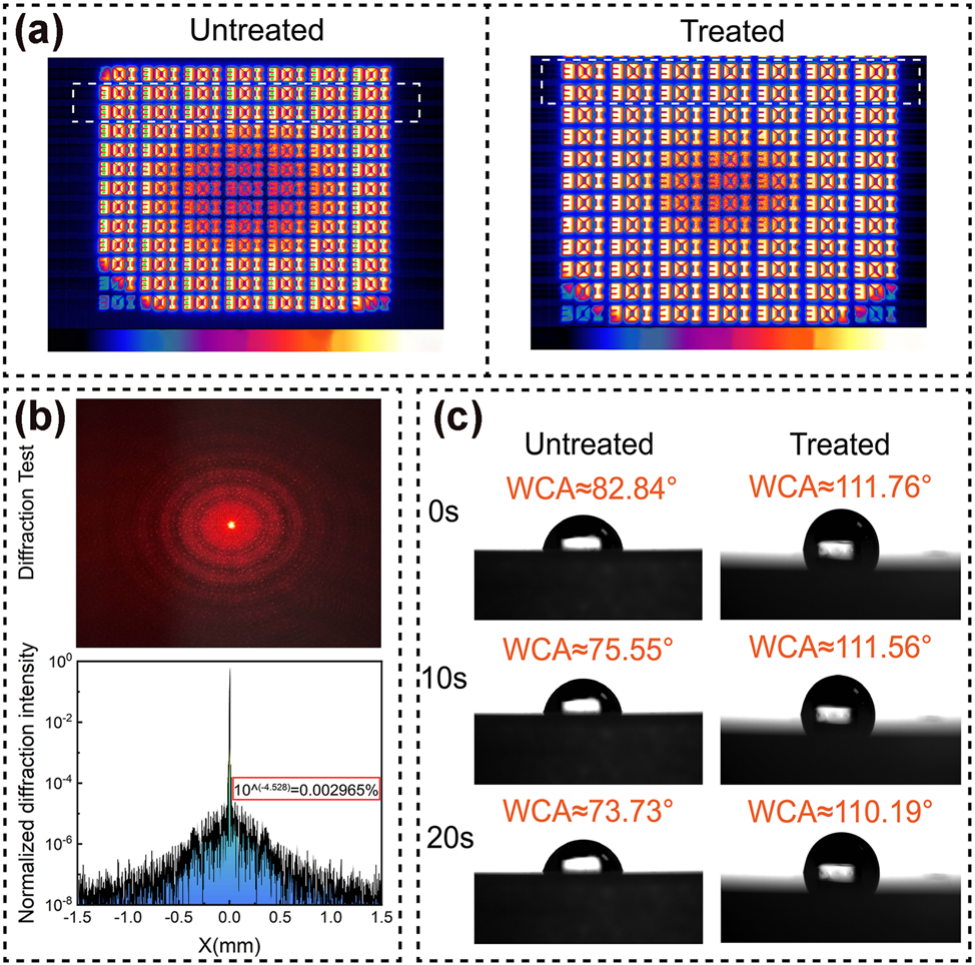
THM low-crosstalk imaging and hydrophobicity parameter testing. (a) Infrared thermal imaging performance of THM and bare ZnS chips was measured at room temperature in a laboratory. (b) Camera-recorded diffraction patterns and program-processed normalized high-order diffraction intensities were characterized. (c) Water droplet contact angles of THM and the bare ZnS chip at different times of 0, 10, and 20 s.

The designed metasurface also functioned as an infrared detection window, exhibiting low crosstalk imaging and hydrophobic capabilities. The low-crosstalk imaging functionality is crucial for infrared optical windows serving as front-end components in photodetectors, to support the detection capability of the backend system, especially in electromagnetic interference environments. To evaluate the interference capability of the THM in infrared imaging, an infrared thermal imaging test was conducted using a blackbody surface light source (radiation temperature of 550 °C ± 3 °C), thermal imaging detector (model: TELOPS SPARK M200, equipped with a standard infrared lens with a focal length to aperture ratio of 3), and custom-made infrared target to parametrically characterize the THM device window performance (experimental setup details provided in Supplementary Material S10). The resulting image is shown in Figure 8(a). Comparative observations demonstrate that images acquired through standard ZnS exhibit pronounced veiling glare artifacts – evidenced by ghosting around the letter ‘E’ contours – while THM samples deliver superior image fidelity with clearly resolved structural details. This enhancement originates from dual synergistic mechanisms: elevated transmittance – where THM achieves >90 % transmission across the 3–5 μm band versus conventional ZnS MS at 70–75 %, directly enhancing photon flux at the detector plane – coupled with stray light suppression through subwavelength antireflection structures that reduce interfacial reflectivity, thereby suppressing multireflection artifacts at their source to mitigate ghost image formation and consequently elevate imaging fidelity. To evaluate the actual optical diffraction energy distribution of the THM, we constructed an optical diffraction test platform, using a 632 nm laser as the light source and an optical setup including a beam expander and diffraction receiving screen, and captured the resulting diffraction patterns with a digital camera system (experimental setup details provided in Supplementary Material S11). Diffraction occurs when a laser beam passes through an expanding lens and irradiates the sample. The resulting diffraction pattern observed on the receiving screen is consistent with the simulation result. Both exhibit a central bright spot surrounded by concentric ring-shaped diffraction structures, confirming good agreement between the simulation and experimental results (in terms of the fundamental diffraction modes). Furthermore, the diffraction image captured using a camera was processed using precompiled programs to extract the intensity distribution and obtain a normalized diffraction energy profile. Figure 8(b) shows that the maximum high-order diffraction energy is low (0.002965 %) and comparable to the simulated value (0.001556 %), confirming that the designed structure exhibits poor optical diffraction characteristics. Hydrophobicity is essential for maintaining optical window performance in complex environments, such as rain or fog. Figure 8(c) shows the measured water contact angles (WCAs) of the untreated ZnS MS sample and THM device. The hydrophobic angle of the untreated ZnS MS sample gradually decreases to 73.73° over time, whereas the THM device consistently maintains a significantly larger hydrophobic angle of approximately 110°. This result demonstrates that in addition to its characteristics of microwave and optical compatibility control and low-diffraction imaging, the THM possesses excellent hydrophobic properties.

## 4 Conclusions

This paper introduces a dual-frequency laser and midwave infrared wide-angle transmission enhancement device that is compatible with electromagnetic scattering regulation and functions as an optically transparent metasurface window. In our strategy, the RCS reduction in the microwave band is achieved through the symmetric cubic phase arrangement of the UHMA, which customizes the wavefront and directs the reflected energy to undergo diffuse scattering. High transmittance over a wide angular range in the 1.42 and 1.7 μm lasers as well as the 3–5 μm mid-infrared bands is achieved through the gradient refractive index and localized field enhancement generated by subwavelength micro-nano structures. As a conceptual demonstration, a 200 × 200 mm^2^ THM sample was fabricated using direct laser writing, lift-off, and etching techniques. Each microwave unit contained over 20 million TMCA, reflecting the scale of processing. The average transmittance achieves >90 % (at 0°–60° incidence angles), covering the optical range of 1.42, 1.7, and 3–5 μm. Moreover, this device facilitates RCS reduction across a broadband range of 9.5–17.5 GHz, thereby decreasing the likelihood of detection under specific conditions. In addition, the THM device exhibited hydrophobic characteristics with a WCA of 110°. In infrared thermal imaging experiments, many microwave-transparent devices experience degradation in imaging quality owing to the diffraction characteristics of their metallic structures. Therefore, THM employs a low-diffraction structure constructed with ultrafine metallic lines, notably decreasing the normalized high-order diffraction intensity, while also reducing the proportion of the metallic structure, enhancing optical transparency, and minimizing crosstalk in imaging. This renders them suitable for practical applications. Multifunctionally compatible transparent metasurfaces have considerable potential for various applications. Finally, because the proposed TMCA adopts a single periodic subwavelength structure, a composite structure with different heights and periods can further expand toward the long-wave infrared region and show improved angular dependence. In addition, more powerful microwave functions can be integrated, which will be investigated in the future.

## Supplementary Material

Supplementary Material Details
