# Supplementary material for: Trans-scale hierarchical metasurfaces for multispectral compatible regulation of lasers, infrared light, and microwaves
Source: Nanophotonics. 2025 Aug 1;14(17):2939–52. doi: 10.1515/nanoph-2025-0224 (PMC12397730; doi:10.1515/nanoph-2025-0224)
Supplement: Supplementary file 1 — Supplementary Material Details [file j_nanoph-2025-0224_suppl_002.docx]

Supporting Information

**Trans-Scale hierarchical metasurfaces for multispectral compatible regulation of lasers, infrared light, and microwaves**

*He Lin, Fuyuan Shen, Zuojun Zhang, Jun Luo, Cheng Huang, Mingbo Pu, Yuetang Wang, Jianping Shi*, Xiaoliang Ma* and Xiangang Luo**

**S1. Theoretical derivation of TMCA equivalent uniform dielectric layer material.**

For a given micron cone unit structure, the equivalent dielectric can be theoretically approximated by the following equation:

$$n_{eff}(h)=\int_{0}^{\text{h}} \left\{f\left(h\right)*n_{Z\text{nS}}^{q}+\left[1-f\left(h\right)\right]*n_{air}^{q}\right\}^{1/q}dh$$

where *f(h)* is the filling factor, which represents the ratio of the corresponding cross-sectional area at the height h of the tetragonal prismatic structure to the area of the cycle; is the refractive index of ZnS MS, which is taken to be 2.45; *n_air_* is the refractive index of air, which is taken to be 1; and *q* is a constant of 2/3. For the tetragonal prismatic structure, the cross-sectional side lengths at the height *h* are a one-time function concerning the height, and there exists the following relationship between its values:

$$a\left(h\right)=\left(1-\left(\frac{h}{H}\right)\right)*S$$

where H is the overall height of the structure and *S* is the length of the bottom side of the structure. Further calculation from equation (2) yields the trend of the filling factor with height, which can be expressed by the following functional relationship:

$$f\left(h\right)=\left(1-\left(\frac{h}{H}\right)\right)^{2}$$

The equivalent refractive index variation of the microcone structure as a function of height can be calculated by substituting the obtained filling factor into Equation (1). Subsequently, performing a definite integral of the curve with respect to height h (h = 1.54 μm) yields an equivalent refractive index of 2.2405 for the structure.

**S2. Unit structure simulation.**

As shown in Fig. S1.a, when the upper layer pattern is rotated clockwise by 45°, the amplitude of the cross-polarization changes slightly. Under the condition of oblique incidence, the amplitude of the cross-polarization of the unit structure gradually narrows in bandwidth as the incident angle increases. When the incident angle reaches 45°, the bandwidth is the narrowest and fails to meet the requirement of being below -10 dB, as shown in Fig. S1.b.


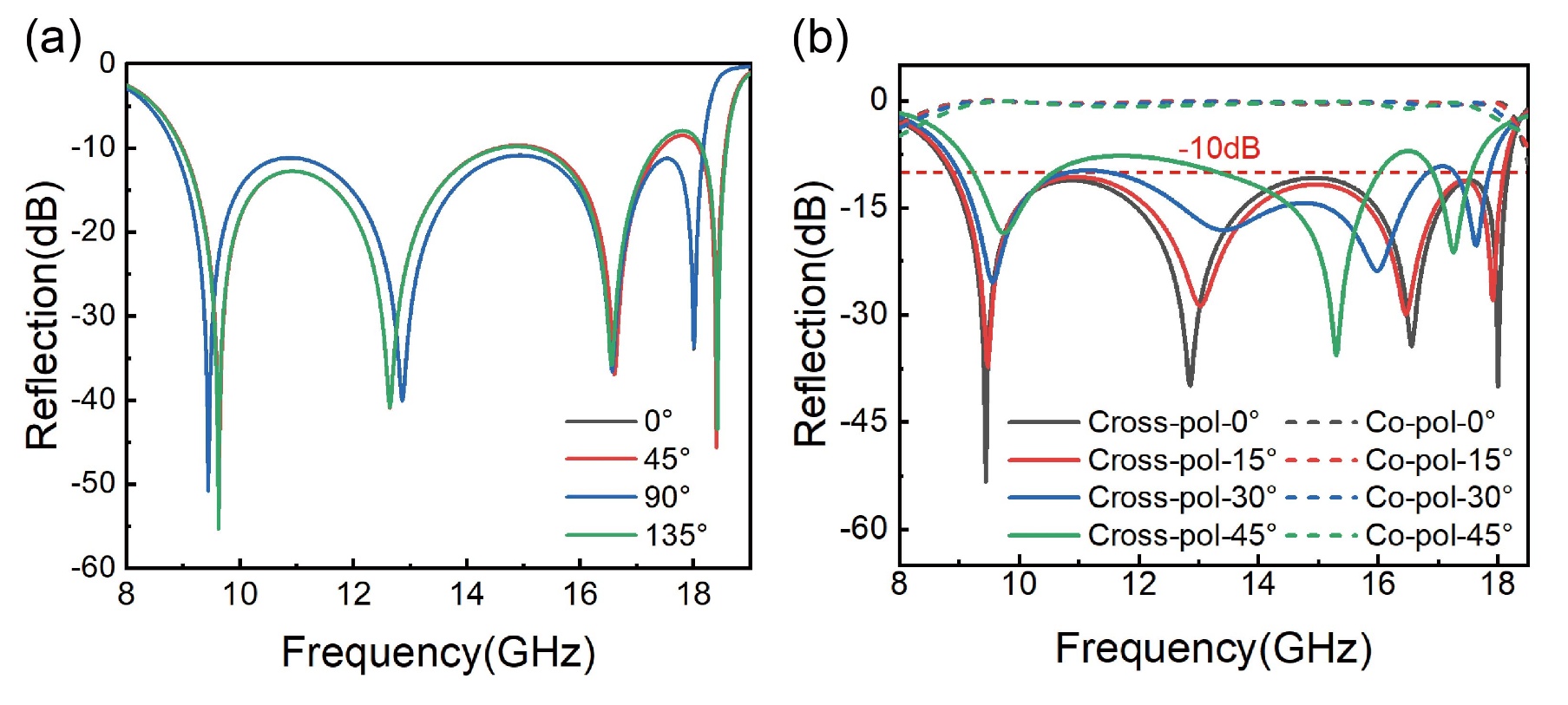


**Fig. S1:** a) the variation in reflection amplitude of the unit structure after rotating clockwise by 45 degrees at intervals, b) the amplitude changes of the unit structure for co-polarization and cross-polarization within the oblique incidence range of 0-45°.

**S3. Modeling the transmittance of metal mesh grids**

From the scalar diffraction theory, the transmittance T of the metal mesh grid is equal to the pore ratio of the grid structure, the light-transmitting portion of the grid divided by the total area of one cycle of the unit structure, which is calculated as follows:

$$T=\frac{S_{t}}{S_{total}}$$

where *S_t_* is the area of the light-transmitting part of the metal mesh grating, and *S_total_* is the total area. However, due to the complexity of the structure of the mesh grid and the direct use of mathematical formulas to calculate the area, the process is redundant and prone to error. Previously, we created a binarized model of the metal mesh grid through discrete matrix programming. The model represents the two-dimensional discrete matrix form of the structure, in which "0" represents the opaque part and "1" represents the transparent part. Therefore, the transmittance of the metal mesh grid can be converted into the form of the ratio of discrete points, it is converted to the ratio of the number of "1"s in the array to the total number of elements., and the expression is as follows:

$$T=\frac{N_{t}}{N_{total}}$$

where *N_t_* is the number of 1's in the discrete matrix and *N_total_* is the number of elements in the matrix. The transmittance is calculated to be about 93% by matrix simulation, i.e., the structure itself produces a transmission loss of about 7%. To verify the correctness of our theoretical calculations, we conducted transmittance testing on the ZnS samples with microwave metal structures that were fabricated. As shown in Fig. S2, the average transmittance of ZnS MS substrate in the mid-infrared band reaches 73.1%, while the measured average transmittance of the crescent-shaped metasurface unit is approximately 67.4%, which is close to the theoretical calculation of 68.3%.


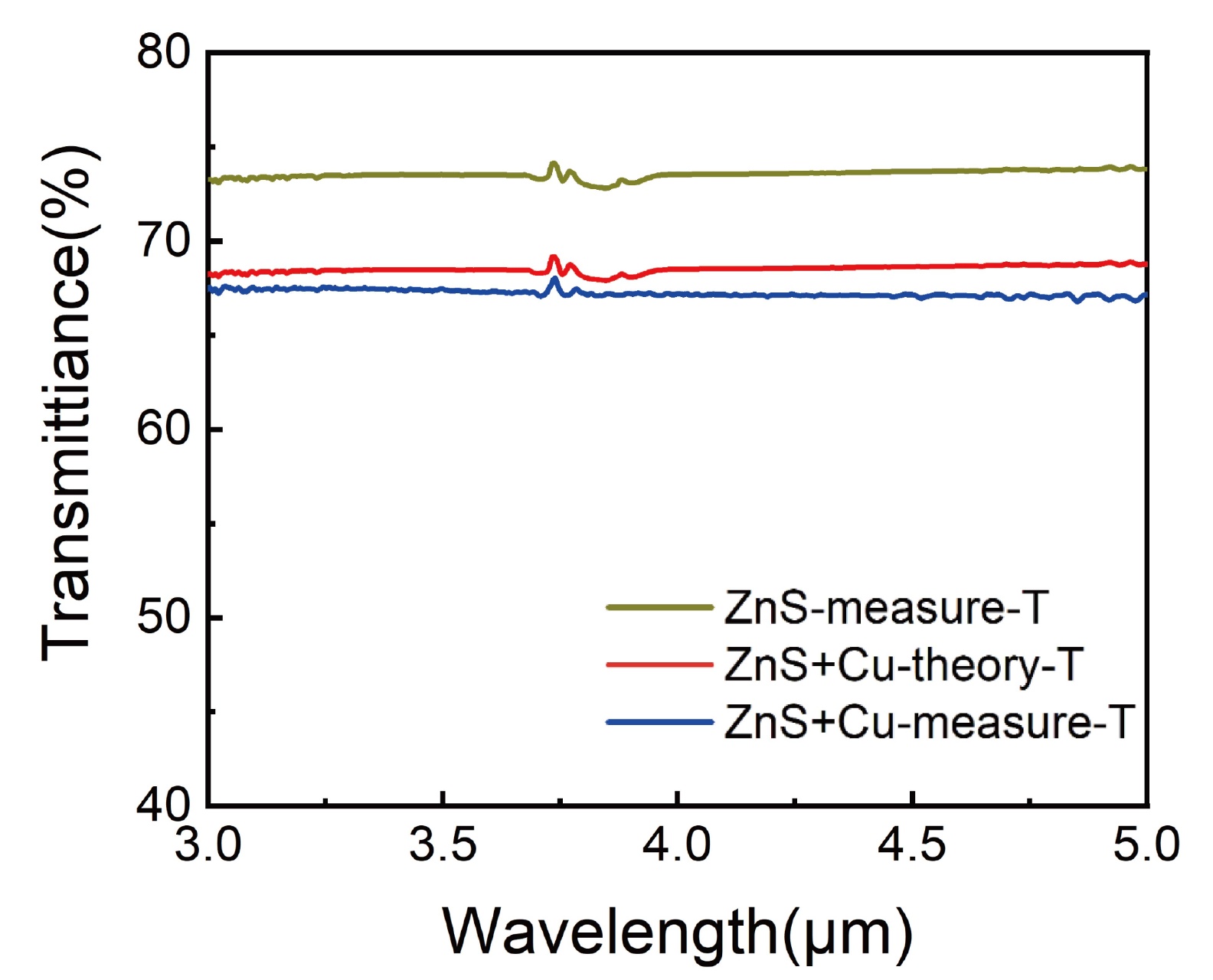


**Fig. S2:** Presents a comparison between the theoretical and experimental transmittance values.

**S4. Simulations for the all-metal mesh metasurface.**

In performing the full-wave simulation, we simulated the RCS reduction effect under the phase distribution at α of 25, 50, 75, and 100, respectively, as shown in Fig. S3.a. It can be seen that when *α* is 50, it can be assumed that the RCS reduction effect is the best in the whole operating band. At 14.4 GHz, the 3D far-field scattering patterns corresponding to different values of α show that when α is 50, the beam is the most uniform, and the low-frequency part meets the basic requirements for RCS reduction. As the value of α increases, the RCS reduction effect in the low-frequency part weakens.


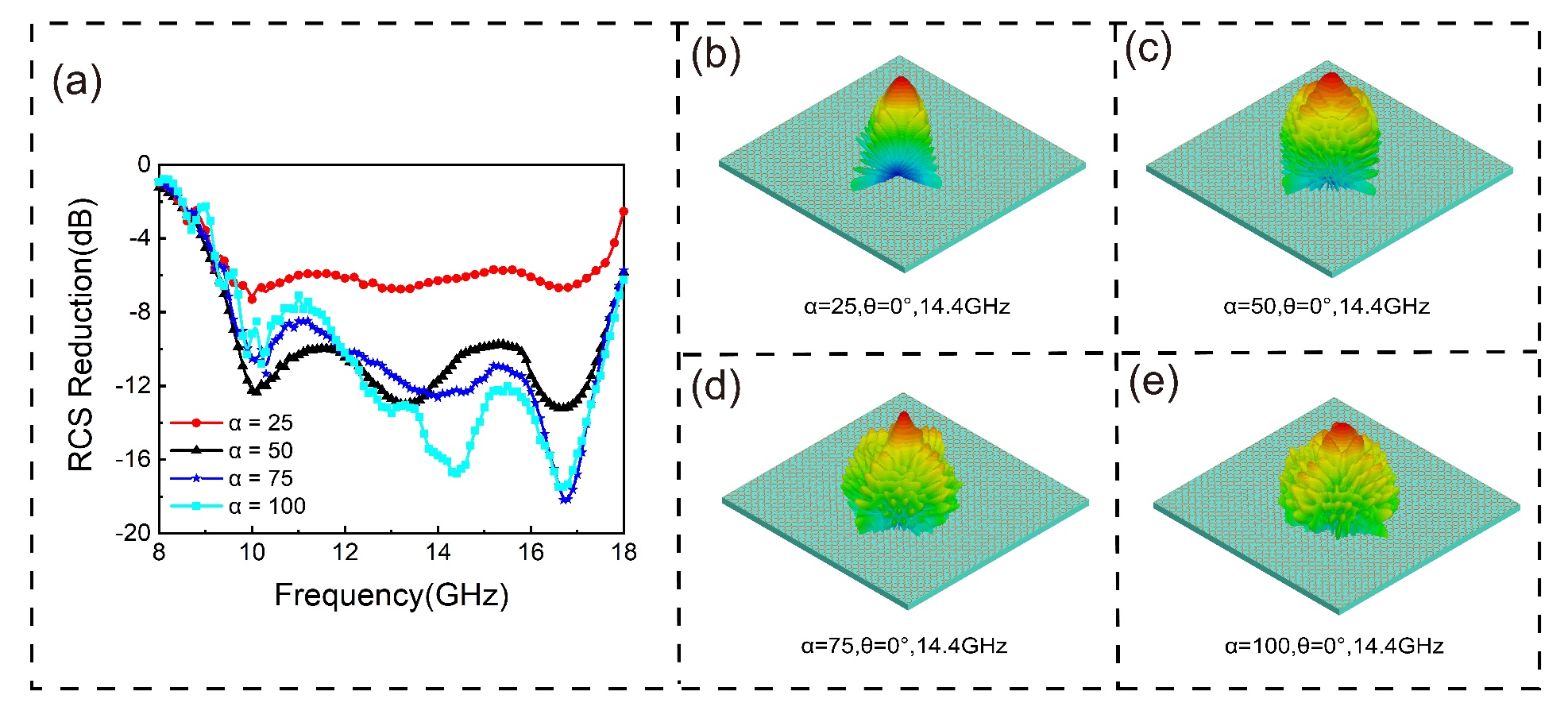


**Fig. S3:** Simulated results of the a) Full-wave simulation results for different *α* values. b-e) 3D far-field scattering diagram of different values of *α* at 14.4 GHz under normal incidence.

We also simulate the case of oblique incidence of electromagnetic waves from 0-45°, as shown in Fig.S4. a. With the increase of the incidence angle, the effective bandwidth of the RCS reduction gradually decreases, and the amplitude fluctuates to some extent, but it still has a certain effective RCS reduction effect within the operating frequency band. Fig. S4. b-e shows the 3D far-field scattering map at 13.2 GHz for *α* of 50 and incident angles of 0°, 15°, 30°, and 45°. When the oblique incidence angle increases to 45°, the RCS reduction bandwidth is the narrowest, while within the range of 0-30°, it exhibits good camouflage performance.


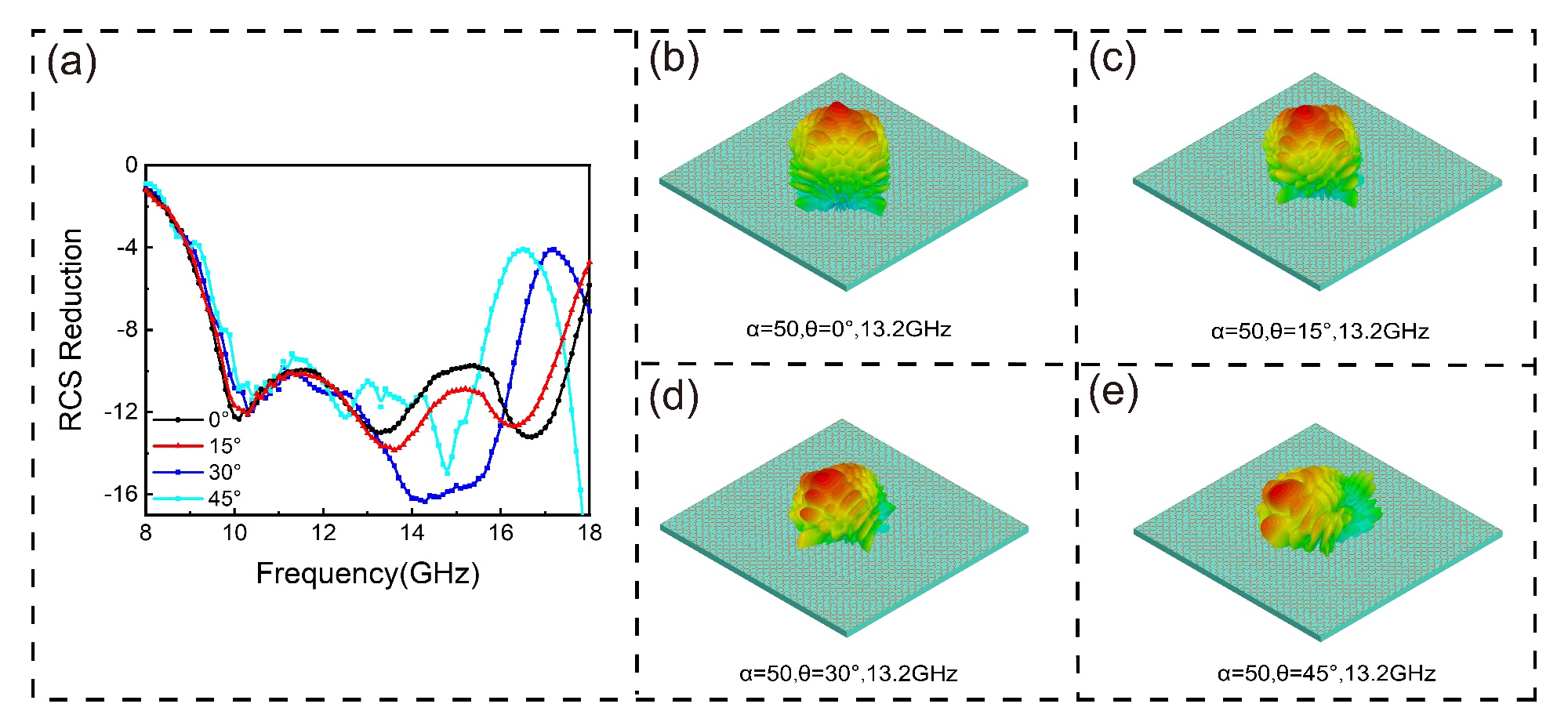


**Fig. S4:** Simulated results of the a) Full-wave simulation results with an oblique incidence of 0-45° b-e) 3D far-field scattering diagram at 13.2 GHz with an oblique incidence of 0-45° when *α* is 50。

**S5. The feasibility of the scheme was analyzed through FDTD simulation.**

Fig. S5 shows the simulation for verifying the feasibility of the proposed scheme. When performing equivalent simulations using FDTD, the boundary conditions in the x- and y- directions were set to periodic, and the boundary condition in the z direction was set to PML, with a plane wave incident. An electric field monitor and a transmission monitor were added. Initially, the basic transmission of the plane dielectric was simulated, as shown by the black curve, after adding the metallic structure on the surface of the dielectric, the transmission decreased, as shown by the green curve. Subsequently, a subwavelength optical anti-reflection structure was added for simulation, resulting in the red curve. It can be observed that the transmission significantly increased, which verifies the feasibility of the design scheme from a simulation perspective.


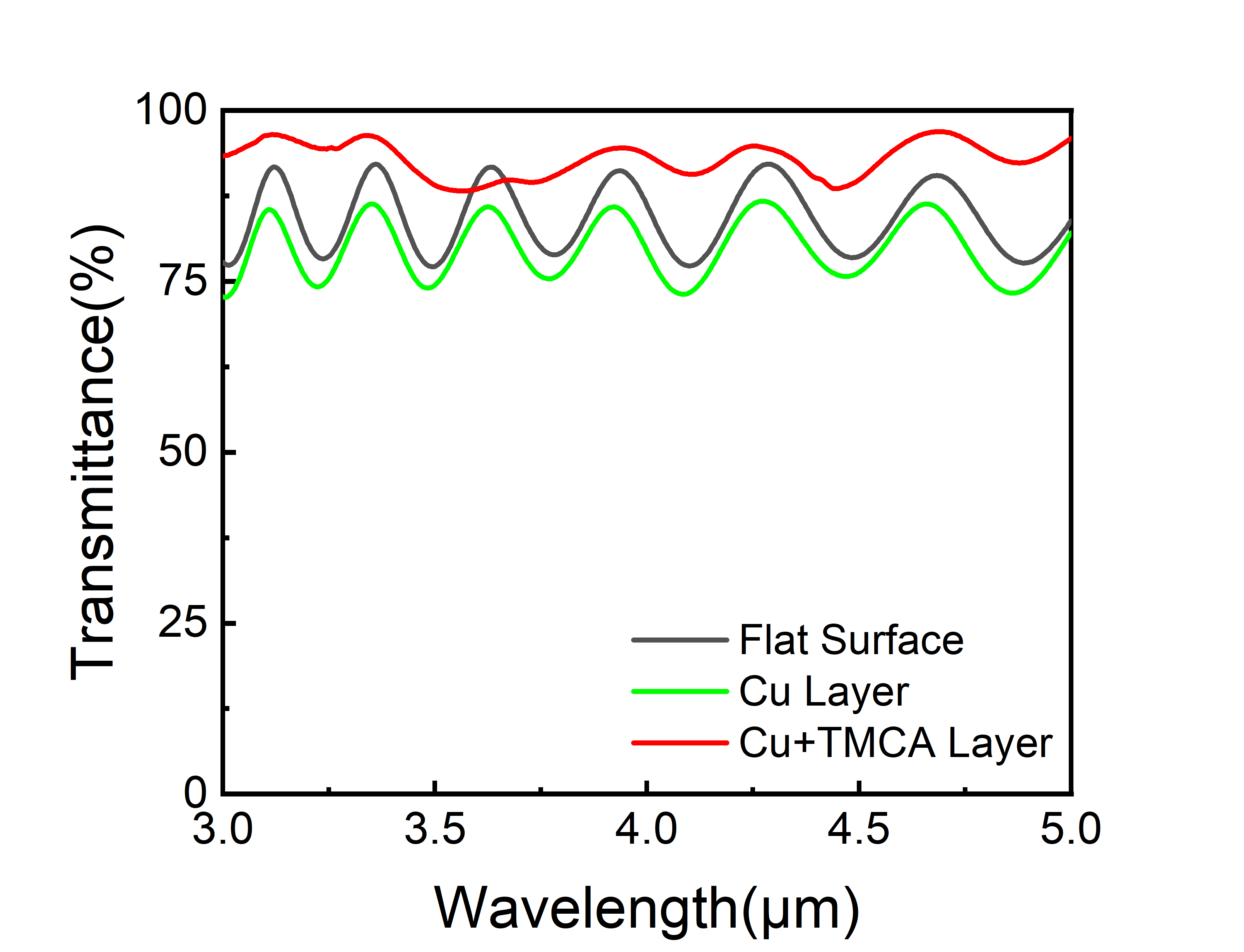


**Fig. S5:** The results of the FDTD equivalent simulation.

**S6. The FDTD simulation of the electric field at the wavelengths of 1.42 and 1.7 μm.**

As the wavelengths are comparable to or smaller than the structural periodic dimensions, multi-diffraction effects occur, resulting in enhanced scattering intensity and higher-order diffraction energy, with a decrease in zeroth-order diffraction energy. Although local field enhancement at subwavelengths diminishes, optimized parameter design ensures transmission enhancement for dual-wavelength lasers.


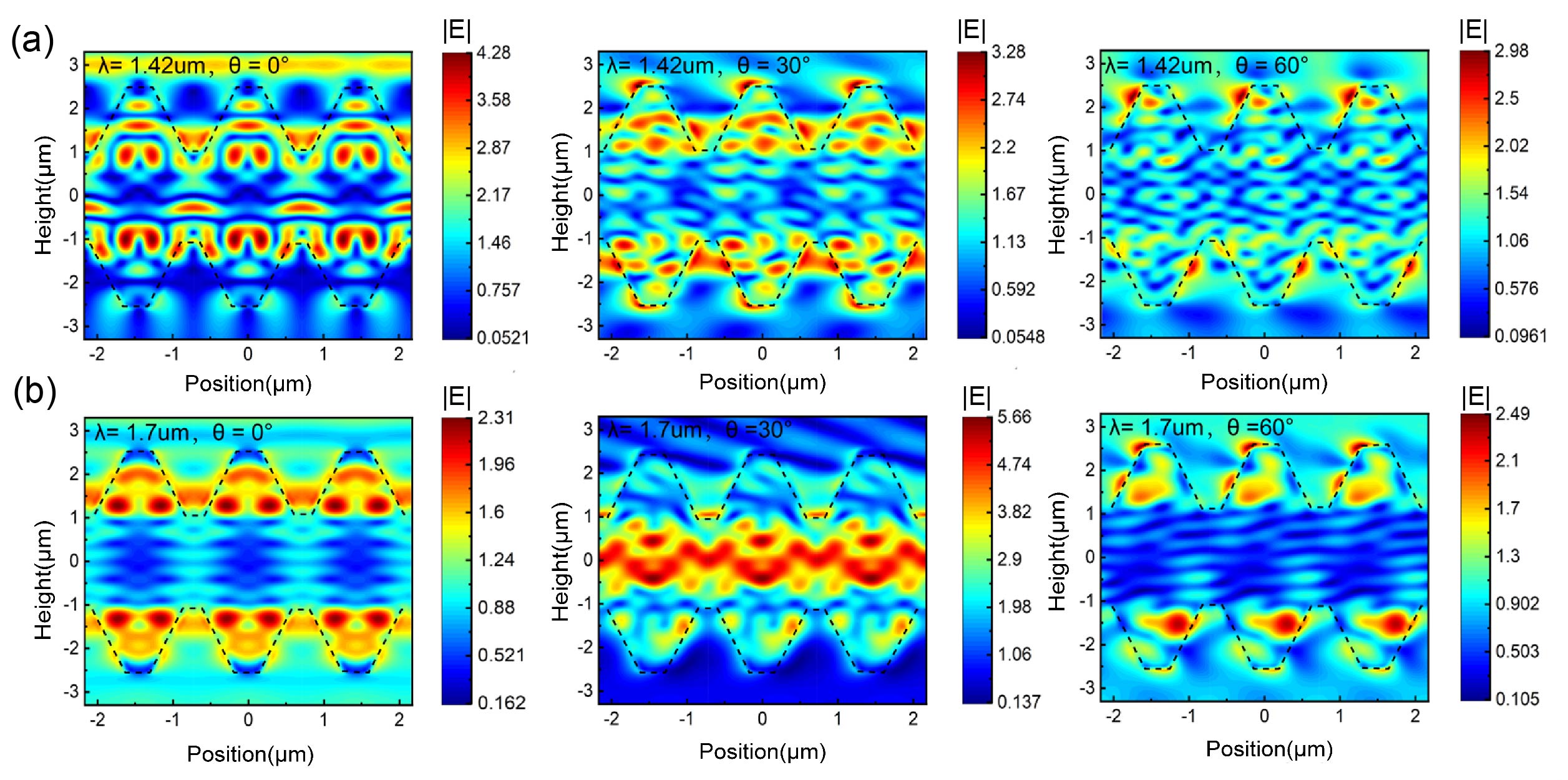


**Fig. S6:** a) shows the electric field distribution at a wavelength of 1.42 μm for incident angles of 0°, 30°, and 60° from left to right. b) shows the electric field distribution at a wavelength of 1.7 μm for incident angles of 0°, 30°, and 60° from left to right.

**S7. TMCA structural parameters are simulated by FDTD**

We used the commercial simulation software FDTD Solution to simulate the designed unit structure, in which the boundary conditions in the X and Y directions are set as Periodic the boundary conditions in the Z direction are set as PML, and the light source is selected to be a plane-wave light source (3-5 μm) incident along the -Z direction, and monitors are set up on the bottom of the structure to monitor the transmission waveforms. Fig. S7 shows the variation of transmittance with the lower base length L, upper base length l, and height h in the 3-5 μm wavelength range. From the simulation results, it can be observed that as the lower base length of the structure increases, i.e., as the duty cycle increases, the transmittance gradually increases. The change in the upper base length has little effect on the transmittance of the structure, while the height of the structure shows that the higher the height, the greater the transmittance. This is attributed to the fact that the change in height determines the variation in the gradient refractive index of the optical antireflection structure.


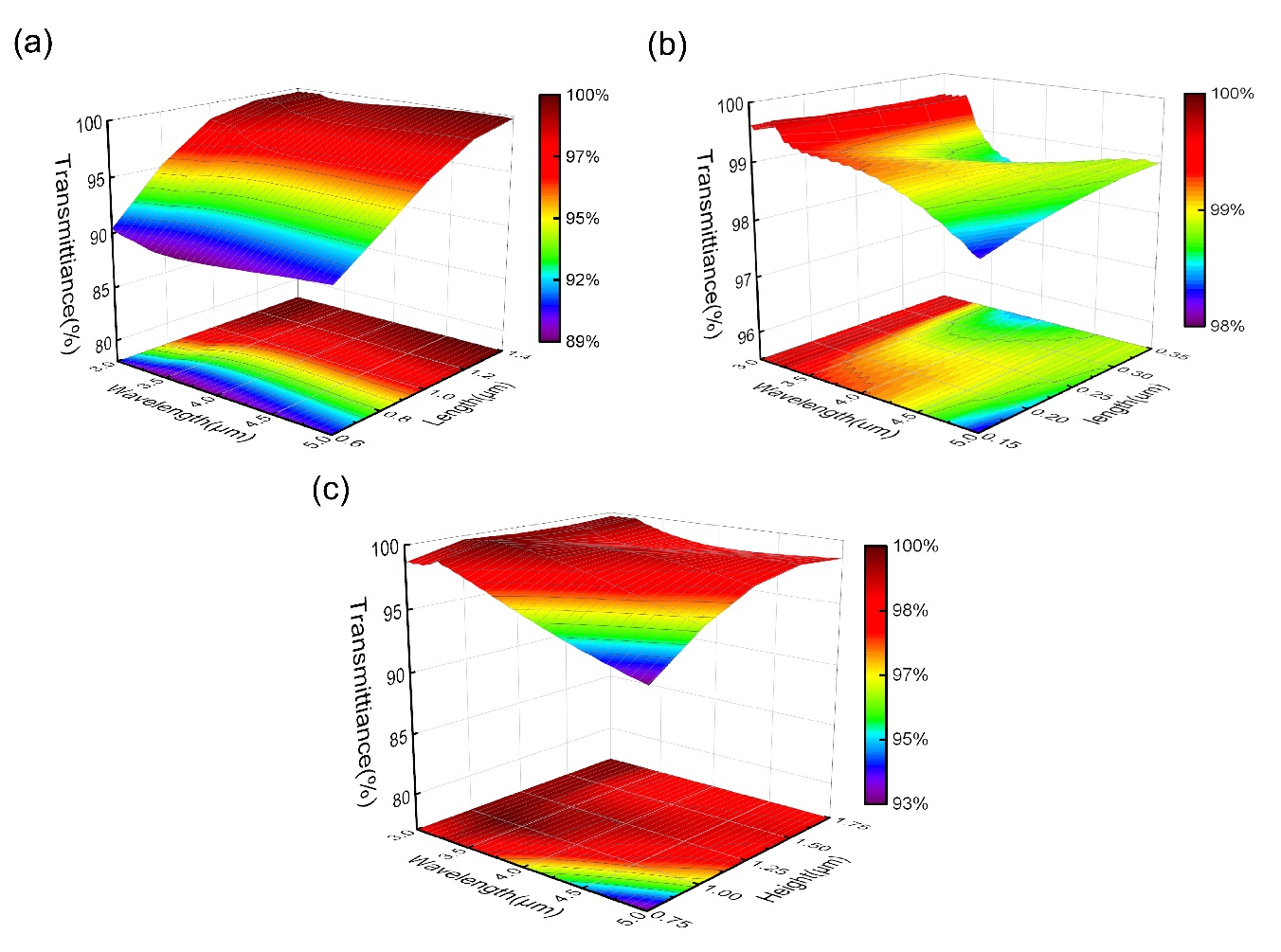


**Fig. S7:** a) shows the variation of transmittance with the lower base length b) shows the variation of transmittance with the upper base length. c) shows the variation of transmittance with the structure height.

**S8. Fabrication**

The lift-off process for substrate fabrication begins with the pre-treatment of the ZnS MS substrate, where a uniform layer of 1.2 μm thick AZ1512 photoresist is applied at 4000 RPM. The exposure equipment used is the Heidelberg DWL66 laser lithography system, which can achieve a minimum line width of 1 μm and has a maximum working area of 200x200 mm2. After photolithographic exposure and development, the photoresist pattern is retained. Next, a thermal evaporation process is employed to deposit a metal thin film. After this, the ZnS MS substrate is immersed in a stripping solution, effectively removing the photoresist and overlying thin film materials, leaving the metal structure layer. Once the metal layer is completed, ZnS MS thin layers are deposited on both sides of the ZnS MS substrate. Then, a 500 nm thick AZ1500 photoresist is spin-coated as the starting material, followed by direct laser writing lithography (DLWL) using a modulated 405 nm laser. The sample is then developed in AZ 400K (AZ Technology) diluted with deionized water in a 3:1 ratio. Afterward, a hard mask is formed using metal sputtering, and the photoresist is removed using ultrasonic-assisted acetone. Finally, TMCA is fabricated through sulfur hexafluoride (SF_6_) plasma reactive ion etching (RIE). As shown in Fig. S8, the morphology in Fig. a indicates that the etching time was too short, while the morphology in Fig. b shows better results. The surface morphology in Fig. c is due to excessive etching time. Taking Fig. b as the reference, the surface profile is modified using the EBL process. Similarly, as the profile modification time increases, the effects shown in Fig. d and e are obtained, with Fig. e showing the best profile control, while Fig. f indicates that the modification time was too long.

**
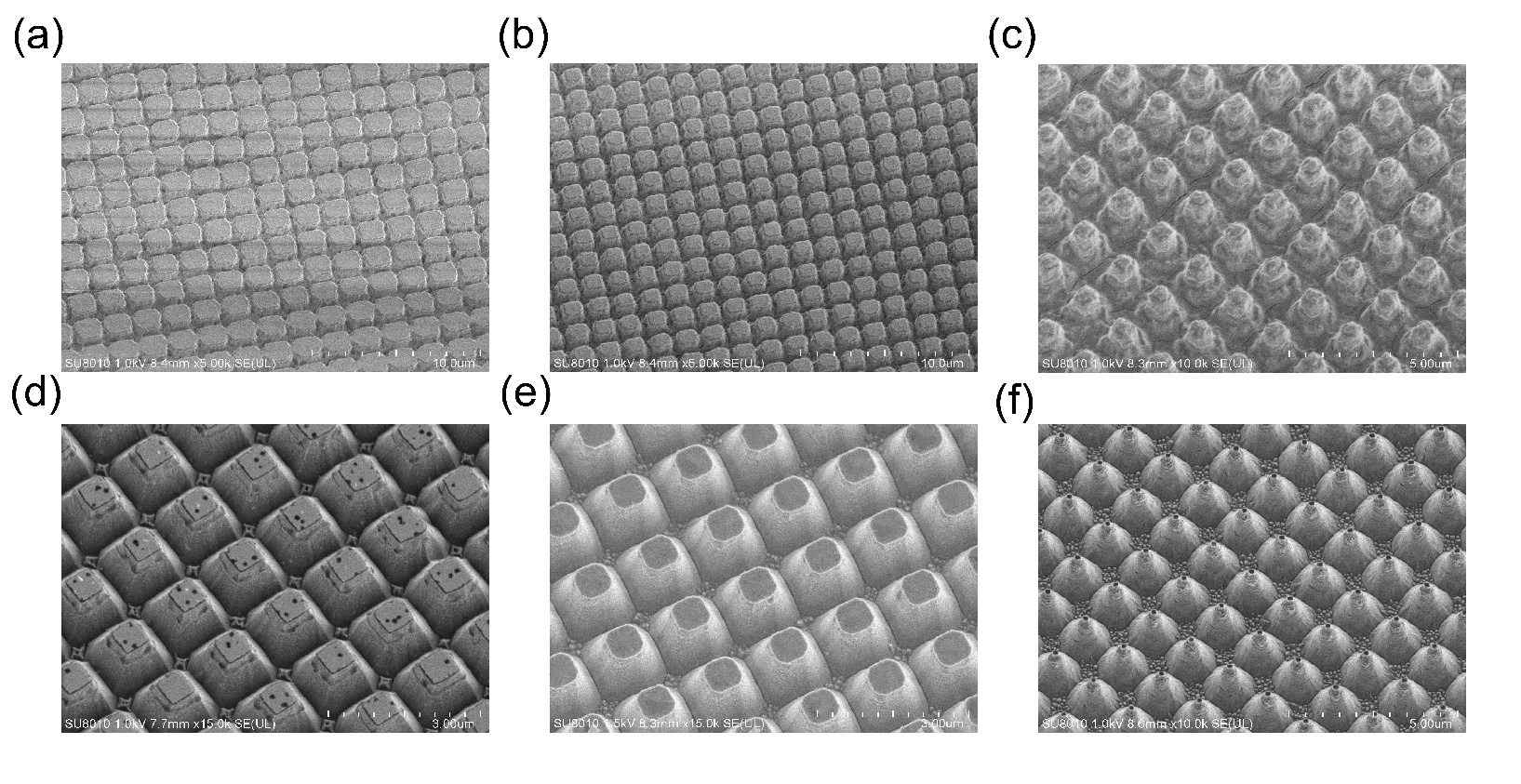
**

**Fig. S8:** The SEM image of TMCA. a-c) represents the surface morphology of TMCA as the etching time gradually increases. d-f) is the effect diagrams of the surface profile modification of TMCA after etching, performed using the EBL process.

**S9. Experimental demonstration for the microwave reflection reduction**

In order to verify the reduction of the Radar Cross Section (RCS) of the metasurface under the illumination of electromagnetic waves in different modes, we have respectively tested the performance curves under the illumination of Transverse Electric (TE) waves and Transverse Magnetic (TM) waves. As shown in Fig. S9, under the illumination of electromagnetic waves with different polarizations, the RCS reduction performance of the Trans-scale Hierarchical Metasurface (THM) remains basically consistent, verifying the polarization independence of the THM, and enabling the camouflage performance within the frequency range of 9.5-17.5 GHz.

**
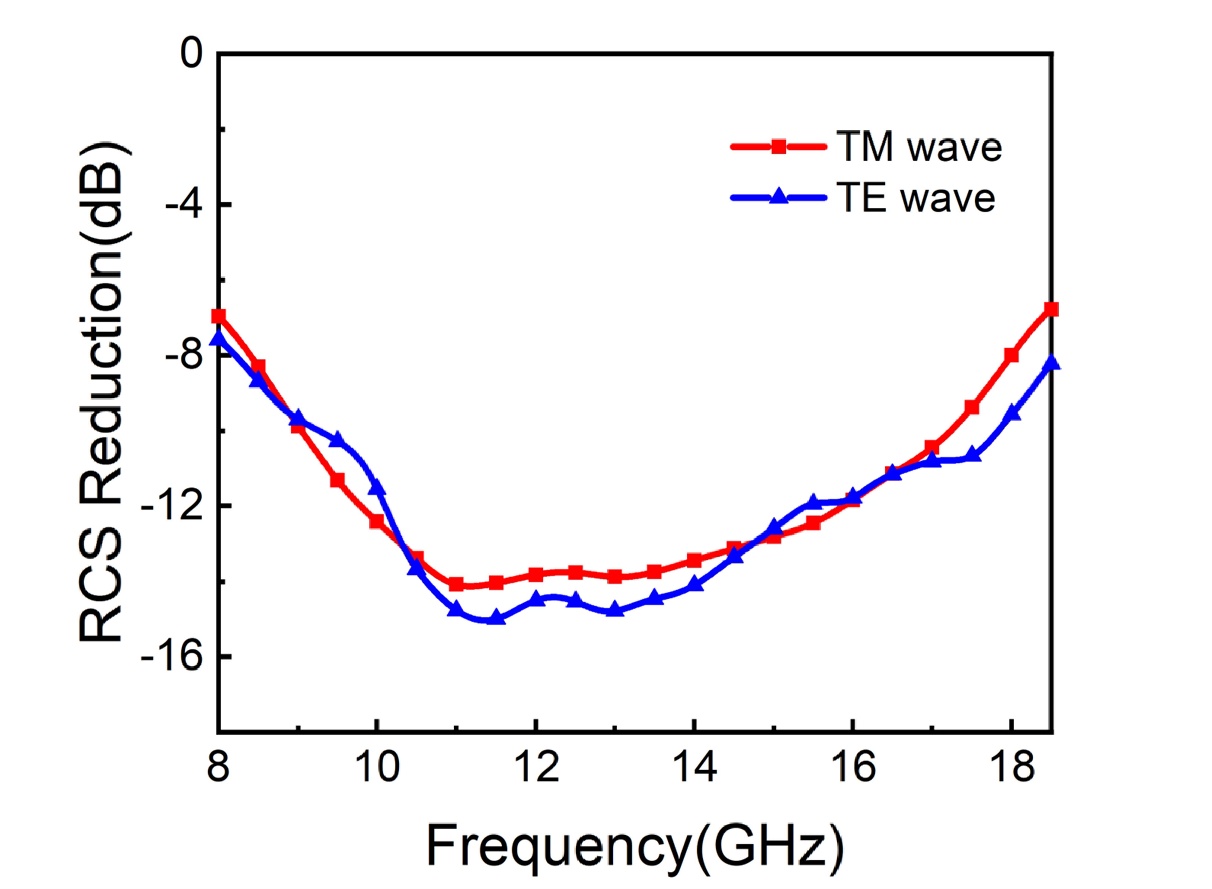
**

**Fig. S9:** TE and TM polarized illumination

**S10. Construction of the infrared thermal imaging experimental platform.**

In order to characterize the infrared imaging interference of the THM sample, we set up an infrared thermal imaging experimental platform. We heated the target using a blackbody radiation source, and then used an infrared camera to collect images through the THM window. By comparing the standard ZnS window with the THM, we obtained the corresponding thermal imaging pictures.

**
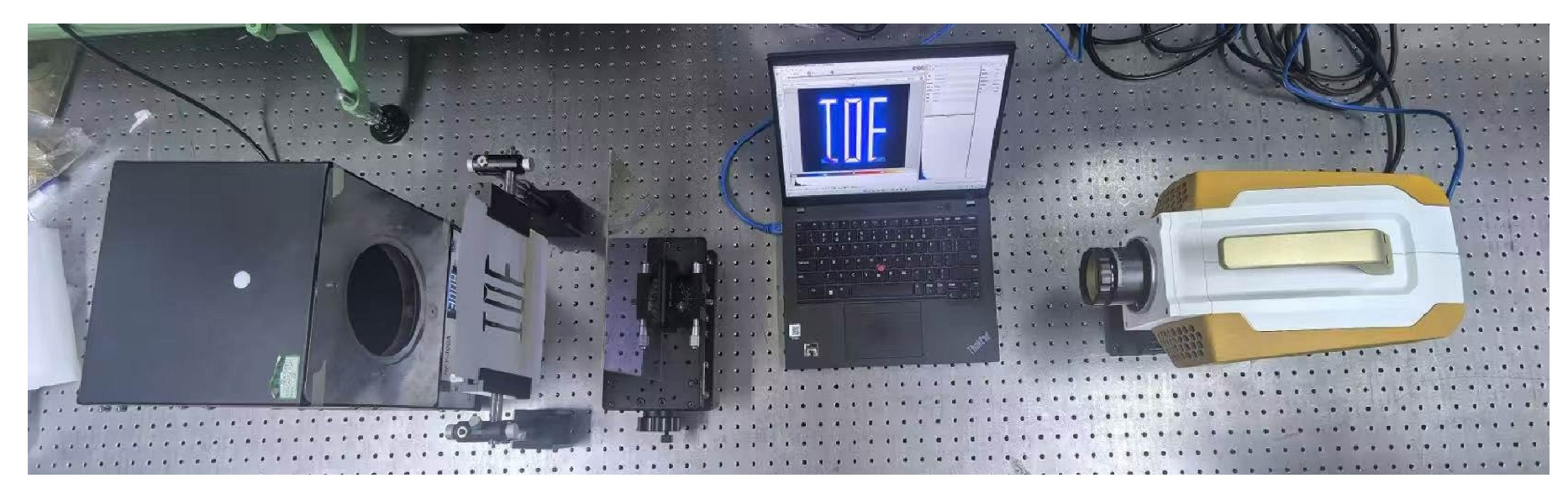
**

**Fig. S10:** From left to right, they are the blackbody radiation source, the target under test, the data acquisition PC, and the thermal imaging detector.

**S11. Building an Optical Diffraction Experiment Platform.**

For experimental validation of both the UHMA's diffraction mode and its normalized high-order diffraction intensity characteristics, a dedicated optical testing system was implemented. This system consists of a 632 nm laser, a beam-expanding lens, the sample under test, and an optical diffraction pattern receiving screen, as shown in Fig. S11. The laser beam emitted by the collimated laser is directed onto the sample through the beam-expanding lens, and the diffraction pattern is projected onto the receiving screen. The diffraction spots generated by the laser passing through the sample can be clearly observed on the receiving screen.


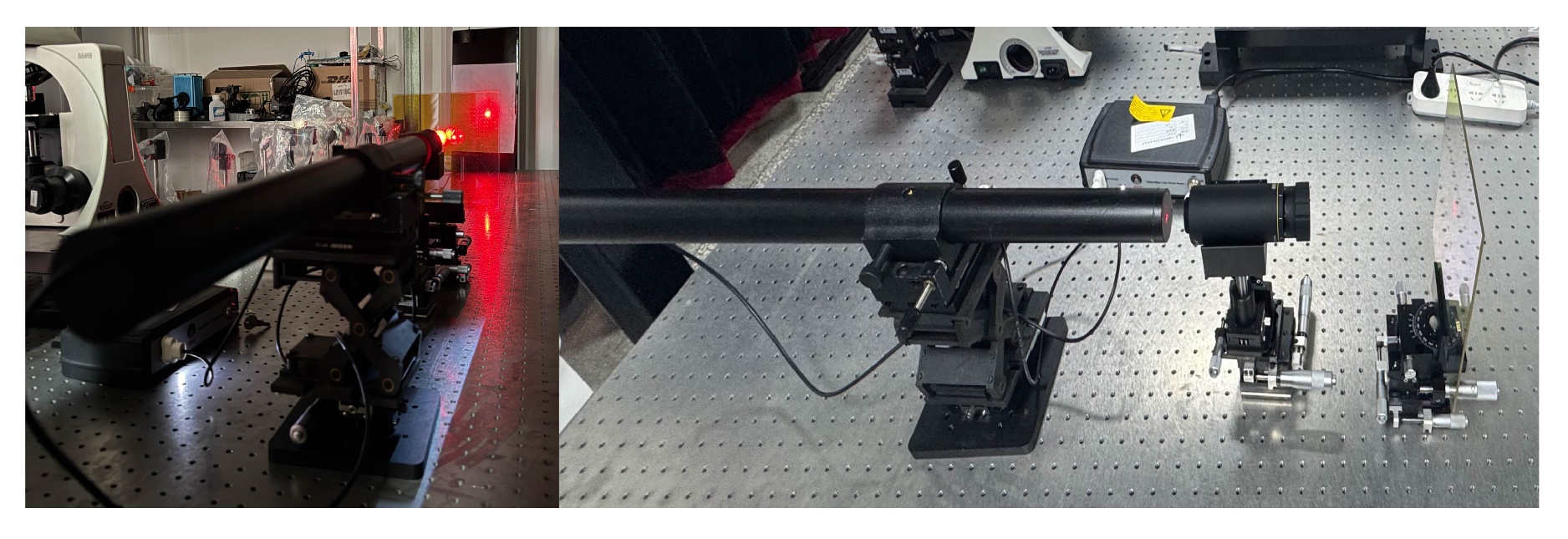


**Fig. S11:** Diffraction Experiment Platform

**S12. The time-domain dynamic distribution of the electric field simulations.**

We incorporated field visualization monitors above and within cross-sectional planes to observe dynamic electric field distributions. As shown in the figure below (double-click image for interactive viewing), we simulated electric field intensity evolution at discrete wavelengths (1.42 and 1.7 μm) and across the 3-5 μm band under illumination angles of 0°, 30°, and 60°. The dynamic results reveal the complete spatiotemporal evolution of light propagation—from initial incidence through structural transmission—clearly demonstrating how subwavelength units channel optical energy into the substrate.  This visualization captures transient field concentration at structural apexes and inter-unit gaps, followed by efficient energy transfer toward the substrate interface. Under normal incidence, the electric field progressively intensifies within structural interstices before migrating directionally toward the substrate interface. At oblique angles, field amplification initially develops along the incident wave vector while propagating inward along subwavelength structural contours as tilt increases. Comparative snapshots of electric field distributions at sequential time intervals directly map optical propagation pathways through the structure, validating synergistic operation between graded-index matching for enhanced transmission and localized field enhancement facilitating energy coupling. Collectively, these dynamic results confirm that field intensification within the structure fundamentally correlates with transmission-mode coupling rather than representing parasitic energy confinement—thus establishing concrete physical underpinnings for the exceptionally high transmittance.


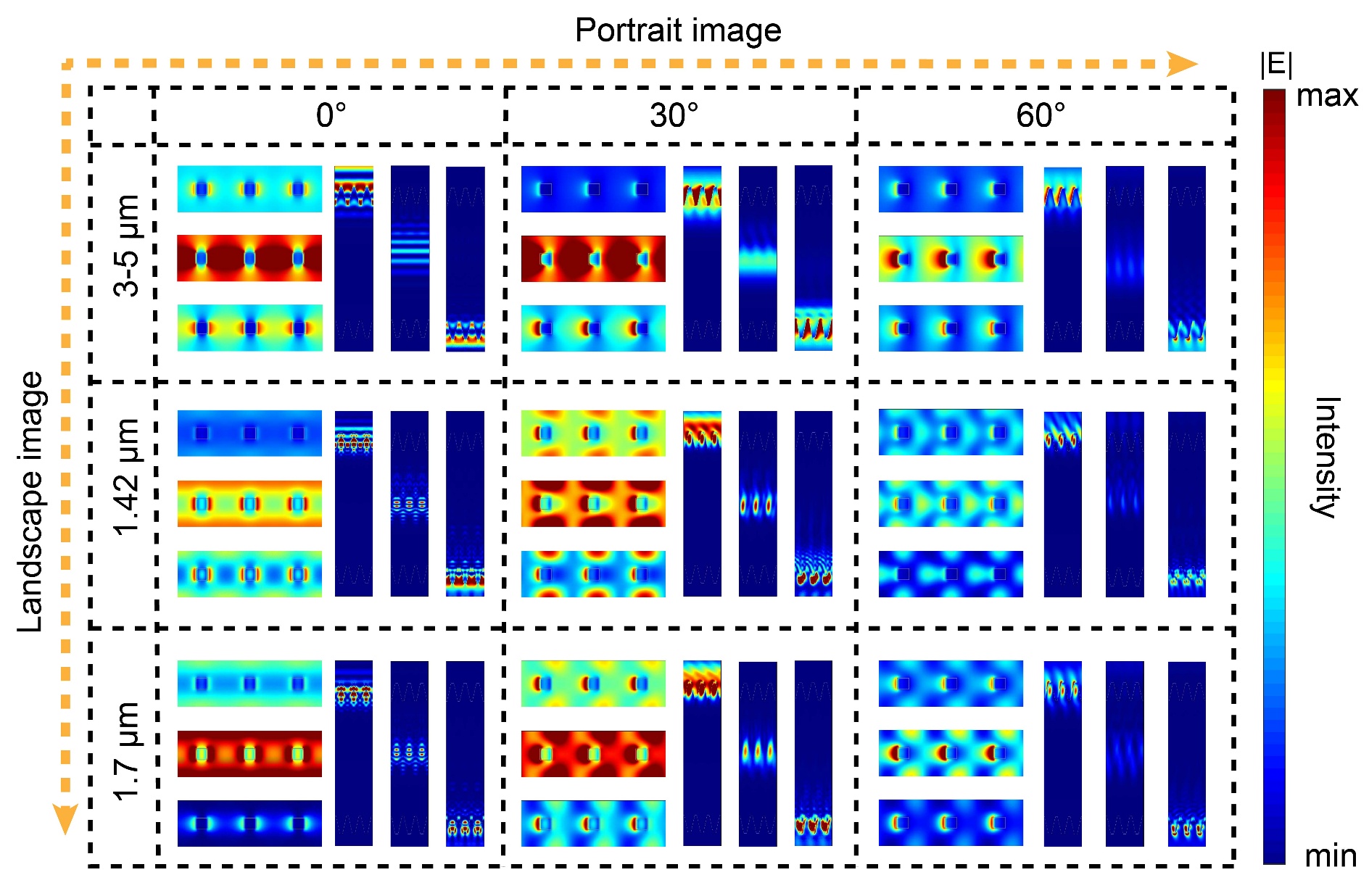


**Fig. S12:** Presents the temporal evolution of electric fields at wavelengths of 1.42 μm, 1.7 μm, and 3-5 μm within both xoy and xoz planes. This visualization reveals dynamic field interactions between subwavelength structures and the substrate following light-wave excitation. Among them, the landscape images show the changes in the electric field at the top layer of the structure in chronological order from top to bottom, while the portrait images display the changes in the electric field of the xoz cross-section in chronological order from left to right.

**S13. Influence of the substrate thickness between subwavelength structures on infrared transmittance and local field enhancement**

The disparity between the simulation spacing (2 μm) and fabricated dimensions (2.5 mm) stems from prohibitive computational demands when modeling full-scale structures given the orders-of-magnitude difference between substrate thickness and microstructural features. Since our primary objective focused on elucidating fundamental structure-property relationships between electric field intensity and transmittance, we employed a simplified model. Following the reviewer's suggestion, we conducted supplementary simulations of micro-pyramid arrays with 10-80 μm spacing under normal plane-wave incidence, implementing periodic boundary conditions (x-, y-directions) and perfectly matched layers (z-direction). Results demonstrate that as spacing increases, the inter-structural electric field remains stable at |E| ≈ 1.63, preserving robust local field enhancement. While transmittance spectra exhibit sinusoidal oscillations, values consistently exceed 95% throughout the tested range. This confirms that array spacing variations exert negligible impact on localized field distribution and minimum transmittance, with the observed spectral oscillations representing predictable Fabry-Pérot-type interference phenomena that do not compromise the physical validity of our model's mechanistic insights.

*
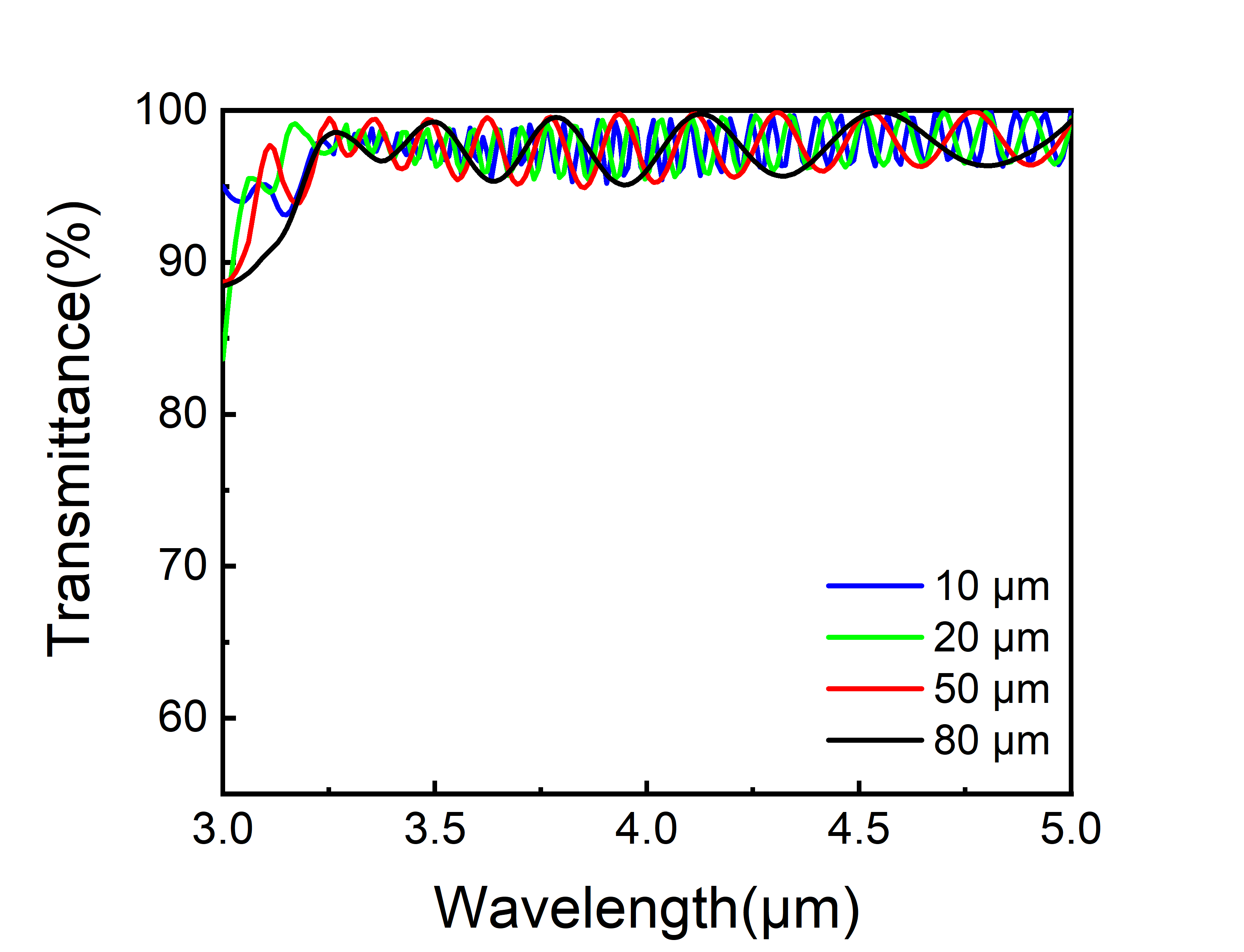
*

**Fig. S13:** Variation of transmittance of micro-nano cones under different spacing conditions.

*
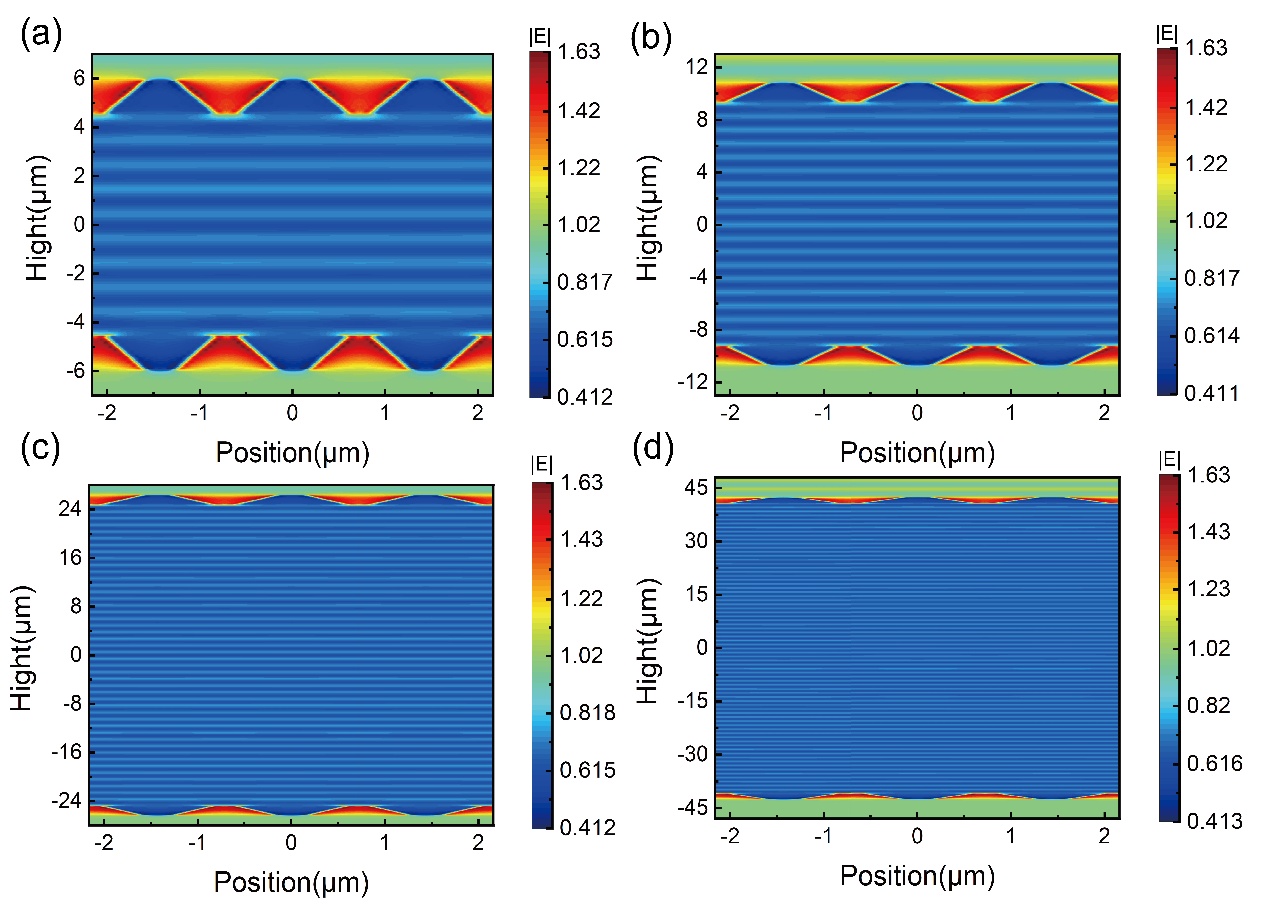
*

**Fig. S14:** (a)-(d) show the variations in the electric field distribution of micro-nano cones under spacing conditions of 10, 20, 50, and 80 μm, respectively.

**S14. The main performance comparison between our work and representative relevant literatures.**

Here we present a comprehensive comparison between the key functionalities and parameters of our proposed multispectral-compatible metasurface (THM) and recent designs reported in the literature, including optically transparent microwave-stealth metasurfaces and optical anti-reflection windows. As clearly demonstrated in Tab. S1, our work exhibits clear advantages in multispectral electromagnetic regulation, wide-angle performance optimization, low diffraction crosstalk in imaging, and multifunctional integration. These strengths provide new design concepts for multifunctional integrated metasurfaces and significantly expand the application scope of metasurface technology.


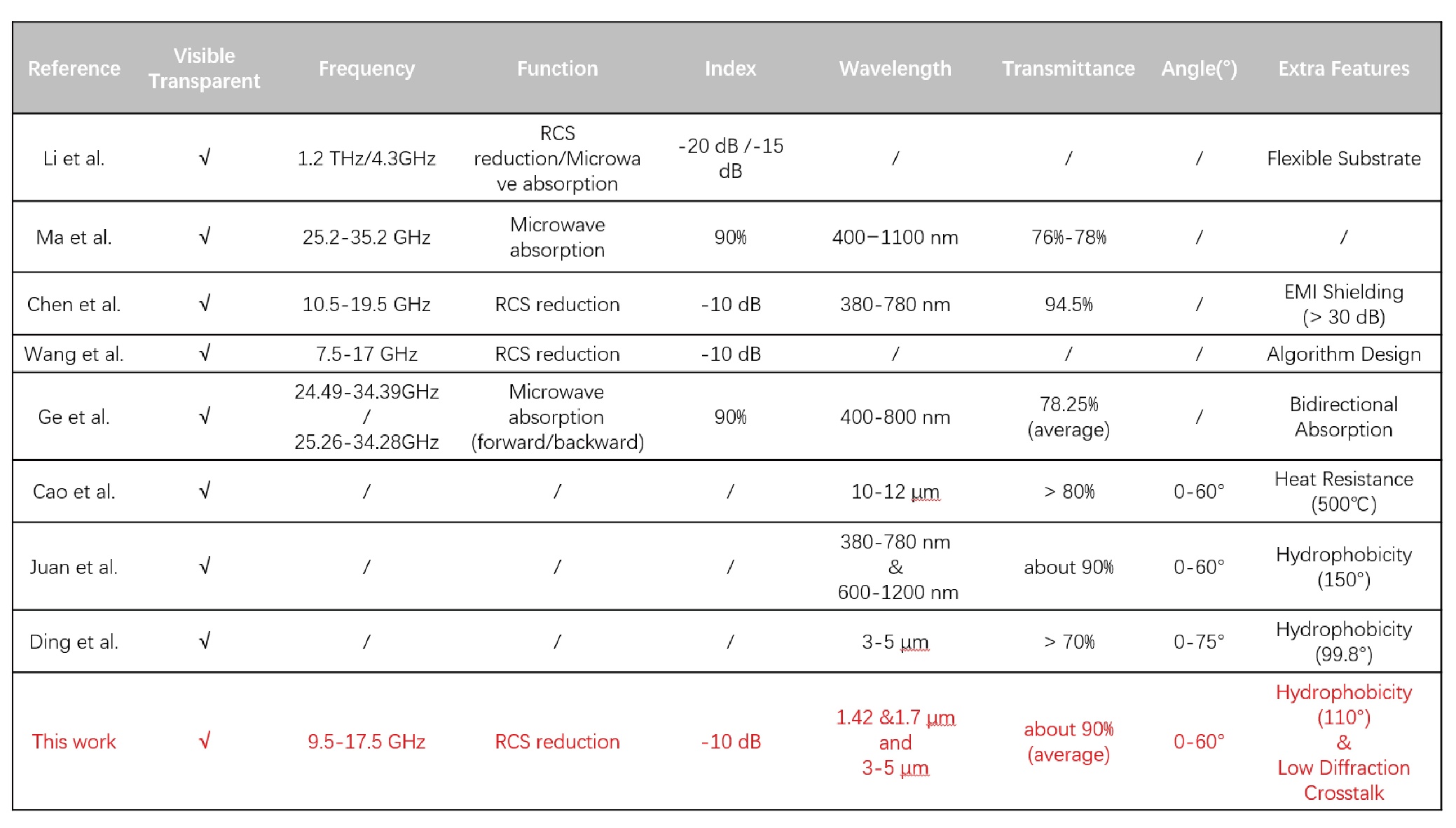


**Tab. S1:** Main parameter list of representative relevant literatures.

Reference in Table S1:

[1] Q. Li, J. Zhang, L. Liu, C. He, and W. Zhu, “Graphene-based optically transparent metasurface for microwave and terahertz cross-band stealth utilizing multiple stealth strategies,” Carbon, vol. 219, p. 118833, 2024, DOI:10.1016/j.carbon.2024.118833.

[2] L. Ma, H. Xu, Z. Lu, and J. Tan, “Optically transparent broadband microwave absorber by graphene and metallic rings,” ACS Applied Materials & Interfaces, vol. 14, no. 15, pp. 17 727–17 738, 2022, DOI: 10.1021/acsami.1c24571.

[3] J. Chen, Y. Wei, Y. Zhao, L. Lin, L. Li, and T. Su, “Transparent and broadband diffusion metasurface with high transparency and high shielding effectiveness using metallic mesh,” IEEE transactions on Antennas and Propagation, vol. 70, no. 7, pp. 5574–5583, 2022, DOI:10.1109/TAP.2022.3145429.

[4] Z. Wang, H. Luo, Y. Cheng, F. Chen, and X. Li, “Design of optically transparent coded metamaterial based on an indiumtin oxide film using deep learning for radar cross-section reduction,” ACS Applied Nano Materials, vol. 7, no. 20, pp.23 558–23 567, 2024, DOI: 10.1021/acsanm.4c03822.

[5] J. Ge, C. Zhang, Y. Zhang, H. Li, J. Wang, R. Jiang, K. Chen, H. Dong, and L. Zhang, “Transparent bilayer ito metasurface with bidirectional and coherently controlled microwave absorption,” Advanced Optical Materials, vol. 11, no. 21, p. 2301268, 2023, DOI: 10.1002/adom.202301268.

[6] H. Cao, Y.-F. Li, G. Wang, H.-Y. Li, D.-R. Sun, Z.-Q. Tang, L.-F. Li, Z.-S. Jin, Y. Yu, X.-Q. Liu et al., “Laser cleaning-assisted femtosecond laser direct writing of diamond an-tireflective microstructures with superhigh transmittance of 94.5% at 10.6 𝜇m,” Small Structures, p. 2400590, 2025, DOI: 10.1002/sstr.202400590.

[7] J. Rombaut, S. Martinez, U. M. Matera, P. Mazumder, and V. Pruneri, “Antireflective multilayer surface with self-cleaning subwavelength structures,” ACS photonics, vol. 8, no. 3, pp. 894–900, 2021, DOI: 10.1021/acsphotonics.0c01909.

[8] Y. Ding, L. Liu, C. Wang, C. Li, N. Lin, S. Niu, Z. Han, and J. Duan, “Bioinspired near-full transmittance MgF_2_ window for infrared detection in extremely complex environments,” ACS Applied Materials & Interfaces, vol. 15, no. 25, pp.30 985–30 997, 2023, DOI: 10.1021/acsami.3c04170.
